# Exploring Conformational Landscapes and Cryptic Binding Pockets in Distinct Functional States of the SARS-CoV-2 Omicron BA.1 and BA.2 Trimers: Mutation-Induced Modulation of Protein Dynamics and Network-Guided Prediction of Variant-Specific Allosteric Binding Sites

**DOI:** 10.3390/v15102009

**Published:** 2023-09-27

**Authors:** Gennady Verkhivker, Mohammed Alshahrani, Grace Gupta

**Affiliations:** 1 Keck Center for Science and Engineering, Graduate Program in Computational and Data Sciences, Schmid College of Science and Technology, Chapman University, Orange, CA 92866, USA; alshahrani@chapman.edu (M.A.); grgupta@chapman.edu (G.G.); 2 Department of Biomedical and Pharmaceutical Sciences, Chapman University School of Pharmacy, Irvine, CA 92618, USA

**Keywords:** SARS-CoV-2 spike protein, Omicron subvariants, ACE2 host receptor, molecular dynamics, protein stability, network analysis, binding pockets, allosteric communications

## Abstract

A significant body of experimental structures of SARS-CoV-2 spike trimers for the BA.1 and BA.2 variants revealed a considerable plasticity of the spike protein and the emergence of druggable binding pockets. Understanding the interplay of conformational dynamics changes induced by the Omicron variants and the identification of cryptic dynamic binding pockets in the S protein is of paramount importance as exploring broad-spectrum antiviral agents to combat the emerging variants is imperative. In the current study, we explore conformational landscapes and characterize the universe of binding pockets in multiple open and closed functional spike states of the BA.1 and BA.2 Omicron variants. By using a combination of atomistic simulations, a dynamics network analysis, and an allostery-guided network screening of binding pockets in the conformational ensembles of the BA.1 and BA.2 spike conformations, we identified all experimentally known allosteric sites and discovered significant variant-specific differences in the distribution of binding sites in the BA.1 and BA.2 trimers. This study provided a structural characterization of the predicted cryptic pockets and captured the experimentally known allosteric sites, revealing the critical role of conformational plasticity in modulating the distribution and cross-talk between functional binding sites. We found that mutational and dynamic changes in the BA.1 variant can induce the remodeling and stabilization of a known druggable pocket in the N-terminal domain, while this pocket is drastically altered and may no longer be available for ligand binding in the BA.2 variant. Our results predicted the experimentally known allosteric site in the receptor-binding domain that remains stable and ranks as the most favorable site in the conformational ensembles of the BA.2 variant but could become fragmented and less probable in BA.1 conformations. We also uncovered several cryptic pockets formed at the inter-domain and inter-protomer interface, including functional regions of the S2 subunit and stem helix region, which are consistent with the known role of pocket residues in modulating conformational transitions and antibody recognition. The results of this study are particularly significant for understanding the dynamic and network features of the universe of available binding pockets in spike proteins, as well as the effects of the Omicron-variant-specific modulation of preferential druggable pockets. The exploration of predicted druggable sites can present a new and previously underappreciated opportunity for therapeutic interventions for Omicron variants through the conformation-selective and variant-specific targeting of functional sites involved in allosteric changes.

## 1. Introduction

A staggering number of structural and biochemical studies of the SARS-CoV-2 viral spike (S) glycoprotein have explored the mechanisms of SARS-CoV-2 infection, showing the key role of the S protein’s dynamics, which are exemplified by functional transitions between distinct functional forms [1,2,3,4,5,6,7,8,9]. Conformational transformations of the SARS-CoV-2 S protein between closed and open S states are exemplified by coordinated global movements of the S1 subunit consisting of an N-terminal domain (NTD), the receptor-binding domain (RBD), and two structurally conserved subdomains, SD1 and SD2, which together determine the structural and dynamic responses of the S protein to binding partners and the host cell receptor, ACE2 [10,11,12,13,14,15]. Biophysical studies provided in-depth characterizations of the thermodynamics and kinetics of the full-length SARS-CoV-2 S trimer, showing that long-range interactions couple coordinated structural changes in dynamic S1 and structurally rigid S2 subunits to modulate population shifts between the open (up) and closed (down) conformations of the RBD, regulating the exposure of the S protein to binding partners and the strength of the S–ACE2 binding [16,17,18]. The rapidly growing number of cryo-EM and X-ray structures of the SARS-CoV-2 S variants of concern (VOCs) in various functional states and complexes with antibodies revealed the remarkable versatility of molecular mechanisms and the diversity of binding epitopes that underlie the binding affinities of S proteins with different classes of antibodies [19,20,21,22,23,24,25,26,27,28]. The cryo-EM structures of the S Omicron BA.1 trimer in the open and closed forms revealed that the dominantly populated conformation is the closed state with all the RBDs buried, leading to “conformational masking” that may prevent antibody binding and neutralization at of receptor binding sites [24]. Another cryo-EM study of the S Omicron BA.1 variants in different functional states showed that the RBDs were tightly packed in the Omicron 3-RBD-down structures, which limits the RBDs’ motions [27]. The cryo-EM structures of the S Omicron BA.1 trimer revealed that the Omicron sites N856K, N969K, and T547K can promote favorable interactions with D658, Q755, and S982 from neighboring subunits, resulting in an increased number of inter-protomer contacts which can confer enhanced stability [28]. Other studies evaluated the thermostability of the S-D614G, S-BA.1, and S-BA.2 protein ectodomains and the stability of their corresponding monomeric RBD constructs via differential scanning fluorimetry (DSF) assays, showing the reduced stability of the BA.1 RBD and the greater rigidity of the BA.2 RBD [29,30,31,32]. Several structural investigations of the Omicron BA.1 variant presented evidence of the enhanced stabilization and thermodynamic preferences for the 1RBD-up open S conformation [33,34,35]. The reported 3.1 Å resolution cryo-EM structure of the S Omicron protein ectodomain [34] and another 3.0 Å cryo-EM structure of the S Omicron protein ectodomain [35] suggested a dynamic shift from the original Wu-Hu-1 strain, which features a mixture of open and closed conformations, to the S Omicron BA.1, which adopts a predominantly RBD-up open form that is predisposed for receptor binding. These studies observed a greater variability of S-BA.2 trimers in unbound and ACE2-bound forms, which may cause the higher transmission and infection rates of the BA.2 Omicron sublineage when compared to the BA.1 variant. Structural and biophysical studies of the RBD-ACE2 complexes showed that that the RBD in Omicron BA.2 is more stable than the BA.1 RBD, while the observed lower temperature for the dissociation of the S-BA.2 trimer indicates that it is more dynamic than the S-BA.1 trimer [36]. The structure of the S-BA.2 trimer complex with human ACE2 revealed a more extensive interaction network in the RBD-ACE2 interface which, combined with the higher stability of the BA.2 RBD compared to the BA.1 RBD, might also contribute to the higher binding affinity of the BA.2 RBD to ACE2 [36].The cryo-EM structures of the S Omicron BA.1 trimers and their comparison with the structures of the G614 S trimers revealed prominent differences in the NTD which contains five point mutations (A67V, T95I, Y144F, Y145D, and L212I), showing that these mutations reconfigured the N-terminal segment and almost all of the surface-exposed loops, including the 143–154, 173–187, 210–217, and 245–260 loops [37].

A recently reported structural and functional analysis of the S-BA.2 protein produced three distinct states representing a closed 3-RBD-down conformation, a 1-RBD-up conformation, and an RBD-intermediate conformation, all of which were also found for the S-G614 trimer [38]. The cryo-EM structures of the S trimers for the BA.1, BA.2, BA.3, and BA.4/BA.5 subvariants of Omicron confirmed that the S-BA.1 trimer is stabilized in an open conformation, while the S-BA.2 trimer exhibits two conformational states corresponding to a closed form and an open form with one RBD in the up position [39]. Thermal stability assays determined that the S-BA.2 trimer was the least stable among the BA.1, BA.2, BA.3 and BA.4/BA.5 variants, revealing a less-compact inter-protomer arrangement [39]. While numerous cryo-EM structures of the S trimers of VOCs provided accurate, high-resolution structural information and described the extent of the opening of various variants, structures alone cannot uncover the intrinsic dynamics and conformational heterogeneity underlying S functions and binding. The hidden dynamic nature of the S trimers was elucidated using a hydrogen–deuterium exchange monitored via mass spectrometry (HDX-MS) [40] which uncovered an alternative open trimer conformation that interconverts with the canonical prefusion structures and can dynamically expose novel epitopes in the conserved region of the S2 trimer interface, providing new epitopes in a highly conserved region of the protein. Another HDX-MS study identified changes in the S dynamics for VOCs, revealing that Omicron mutations may preferentially induce closed conformations and that the NTD acts as a hotspot of conformational divergence driving immune evasion [41]. Cryo-EM and crystal structures for seven NTD-directed neutralizing antibodies in complex with either a SARS-CoV-2 S trimer or an isolated NTD showed that all these antibodies targeted an NTD-antigenic supersite, leading to conjecture that all neutralizing NTD antibodies might recognize and remodel this dynamic site, creating binding-induced cryptic pockets in this region [42]. A systematic comparison of the efficacy with which human monoclonal antibodies cover the seven identified epitope classes of the SARS-CoV-2 RBD against the BA.1, BA.2, and BA.3 variants confirmed the enhanced immune evasion profile of the BA.2 variant, which may arise from the conformational variability of the S trimer [43,44]. Cryo-EM tomography experiments unveiled a complex landscape of the S protein, capturing not only dynamic states but also partially folded, intermediate S states on the pathway to membrane fusion [45]. Together, a significant body of experimental S trimer structures for the BA.1 and BA.2 variants revealed the considerable plasticity of both the closed and open forms and the emergence of dynamic intermediate states, particularly in the BA.2-variant trimers. There is no consensus on how Omicron variants can affect thermodynamic preferences for open or closed states, thus highlighting the elusive nature of the dynamic spike equilibrium and the allocation of binding sites that can be modulated via various mutations.

There has been a significant effort toward and a surging interest in identifying cryptic dynamic binding sites in the S protein’s structures. Cryptic binding pockets are typically either absent or undetectable in unliganded (apo) protein structures but could become more structurally visible and dominant due to ligand–protein binding or because of dynamic changes that remodel and stabilize these binding sites [46]. Cryptic binding pockets can be druggable and often form functionally important allosteric binding sites distant from known functional sites, thus unleashing the potential for the sequestering of dynamic protein states and eliciting functional effects through conformation-specific allosteric targeting via potent and selective modulators [46]. Crystal and cryo-EM structures often represent only a fraction of probable conformational states, and some allosteric binding sites are not readily apparent from these static structures. A large-scale analysis of crystal structures harboring validated cryptic sites showed that the conformational changes associated with the emergence and stabilization of cryptic sites may occur via loop movements and interdomain motions near flexible protein regions [47,48]. It was observed that unbound protein structures with these hidden pockets often have binding hotspots in flexible regions neighboring the cryptic site that can be exploited by the bound ligand [47].

Strikingly, several cryo-EM structures have uncovered cryptic pockets in the RBD and NTD regions of the S protein which serve as potential druggable epitopes [49,50,51,52,53,54]. A highly conserved cryptic epitope in the RBD was discovered in one of the early structural studies [49]. The cryo-EM structure of the S protein’s complex with linoleic acid (LA) revealed an allosteric binding pocket in the S-RBD (often referred as the LA pocket) that is modulated through the opening of a gating helix and allosterically induces reduced levels of S binding in the presence of LA [50,51,52]. A highly conserved cryptic epitope at the S trimeric interface enables a broad antibody recognition of Omicron variants, suggesting a possible mechanism for antibody neutralization via inducing the disassembly of the S trimer [53]. A cryptic binding pocket in the NTD was revealed in structural studies, showing that a polysorbate (PS) detergent molecule could occupy this site when the detergent was present in the formulation of the immunogen [54]. Cryo-EM and X-ray crystallography studies discovered that this cryptic site on the NTD binds the tetrapyrrole products of the heme metabolism, biliverdin and bilirubin, with nanomolar affinity blocking the access of antibodies to their NTD epitopes on the S protein [55]. The cryo-EM structure of a vaccine-induced antibody discovered a novel neutralizing epitope on the NTD that is defined by a cryptic, hydrophobic NTD cavity that binds the heme metabolite biliverdin [56]. Moreover, due to the generally conserved and yet plastic nature of the epitope, the antibody maintains binding to VOCs, including the Omicron BA.1 variant [56]. Other recent studies highlighted the conformational plasticity of the NTD regions in which mutations/deletions not only change the architecture but also alter the surface properties, leading to the remodeling of the binding pockets, major antigenic changes in the NTD supersite, and a loss of antibody binding [57]. These studies supported the notion that the NTD can serve as an adaptable antigenic surface on the S protein capable of unlocking and redistributing cryptic binding pockets which may enable the virus to divert the host’s immune responses away from the RBD regions [40].

Computer simulations provided important atomistic and mechanistic advances for understanding the dynamics and function of the SARS-CoV-2 S proteins [58,59,60,61,62,63,64]. Conformational dynamics studies using cryo-EM showed that the closed ground S state may be in equilibrium with a 1 RBD-up form that is trapped by ACE2 binding, leading the conformational landscape of the S trimer to shift and enhancing conformational plasticity in the S1 subunits [61]. Large-scale adaptive sampling simulations of the viral proteome captured the conformational heterogeneity of the S protein and predicted the existence of multiple cryptic epitopes and hidden allosteric pockets [62]. Replica-exchange molecular dynamics (MD) simulations examined the conformational landscapes of full-length S protein trimers, discovering transition pathways via inter-domain interactions, hidden functional intermediates along open-closed transition pathways, and previously unknown cryptic pockets that were consistent with FRET experiments [63,64]. The cryo-EM MetaInference (EMMI) method modeled conformational ensembles by combining simulations with cryo-EM data, revealing the intermediate states in the opening pathway of the S protein and discovering potentially druggable novel cryptic sites near the RBD recognition site [65]. Our recent studies combined multiscale simulations with coevolutionary analyses and network-based modeling to reveal that the S protein can function as a functionally adaptable, allosterically regulated machine that exploits the plasticity of allosteric centers to fine-tune the response to antibody binding [66,67,68,69,70].

Computer-simulation-based mapping of full-length models of the S protein in the presence of benzene probes reproduced experimentally discovered allosteric sites and identified several novel and potentially druggable pockets on the S protein’s surface [71]. These pockets are hydrophobic and are characterized by well-conserved surfaces and their spatial proximity to regions of functional relevance in viral assembly. The dynamic conformational ensembles of proteins may unveil cryptic sites otherwise hidden in the crystal structures. For example, the positive allosteric modulators (PAMs) of the M1 muscarinic acetylcholine receptor (mAChR) showed exquisite selectivity by occupying a dynamic pocket which was absent in existing crystal structures but revealed in MD simulations [72]. Conducting a Gaussian-accelerated molecular dynamics (GaMD) sampling of the conformational space identified cryptic pockets within the main protease of SARS-CoV-2, including previously unknown druggable pockets which are distant from the active site [73]. There has been considerable progress in developing efficient and robust machine learning (ML) tools for the rapid screening and identification of putative ligand binding sites and allosteric pockets [74,75,76,77,78,79]. Recent studies revealed that cryptic binding pockets emerging from conformational ensembles can be identified in large proteins where these pockets are hidden in experimentally determined structures [80,81]. Nonetheless, the enhanced computational power and accuracy of ML-based binding-pocket predictors have not been applied to probe conformational ensembles for the systematic screening and classification of binding pockets in order to determine and differentiate functionally significant cryptic pockets and allosteric binding sites. In one such study, a general computational strategy was applied and validated in the identification of cryptic pockets to the interferon inhibitory domain (IID) of the Ebola virus viral protein 35 (VP35) [82]. This pipeline included adaptive sampling simulations and Markov state model (MSM) methods, together with a machine learning algorithm, DiffNets, which is a supervised autoencoder architecture that can differentiate between structural ensembles [83]. By combining MD simulations, Markov state models (MSMs), and a novel MSM-based approach to aggregating docking results across structural ensembles, a recent study identified cryptic pockets targeted by allosteric myosin-II; this functional pocket is always closed in ligand-free experimental structures [84]. The cryptic binding sites were uncovered in AlphaFold-predicted ensembles of PPM1D phosphatase, and a neural network trained to evaluate the quality of docked poses predicted that this site is the most likely binding mode for the allosteric inhibitors of PPM1D [85].

MD simulations of unbound or ACE2-bound RBD conformations combined with a pocket analysis and a prediction of druggability identified several promising druggable sites, including one located between the RBD monomers [86]. Recent functional studies identified that the metabolite of fenofibrate (fenofibric acid or FA) destabilized the RBD protein and significantly reduced infection rates in vitro by inhibiting ACE2 binding [87]. MD simulations and an energetic analysis suggested that FA induces a conformational change in the RBD by stabilizing a potential cryptic binding site on the RBD [88]. The reversed allosteric communication approach, which is based on the premise that in proteins, allosteric signaling is bidirectional and can propagate from an allosteric to an orthosteric site and vice versa, has been used for the characterization of binding shifts in protein ensembles and the identification of cryptic allosteric sites [89,90,91]. A network-based adaptation of the reversed allosteric communication approach identified allosteric hotspots and RBD binding pockets in RBD-ACE2 complexes of the Omicron variant [92]. The integration of computational and experimental studies enabled the discovery and validation of the cryptic allosteric site located between subdomains of the S protein, with several targeted compounds showing characteristic binding and anti-virus activities [93,94].

Despite recent progress in the detection of cryptic binding sites, these approaches are often cumbersome and time-consuming, presenting a considerable challenge for the systematic screening and characterization of functionally relevant cryptic pockets and potential allosteric binding sites. This is particularly important for studies of conformational dynamics and pocket analyses of the SARS-CoV-2 S protein and various Omicron variants. The mechanisms through which perturbations induced by Omicron mutations alter the conformational landscape of the S protein are complex, and the effects of the mutation-induced dynamic and structural changes on binding epitopes and the distribution of binding sites remain unknown, including the principles guiding the dynamic formation of cryptic binding pockets. Concurrently, there has been an increasing interest in identifying druggable sites in the S2 subunit as most recently developed broad-spectrum fusion inhibitors and candidate vaccines can target the conserved elements in the S2 subunit [95,96].

In the current study, we perform a systematic, comparative analysis of conformational dynamics and allostery in the multiple open and closed functional states of the S trimers of the BA.1 and BA.2 Omicron variants. By using a combination of coarse-grained and all-atom molecular dynamics (MD) simulations and a dynamics network analysis, we identify the distribution of emerging binding pockets in the S trimers for the BA.1 and BA.2 variants on a large scale. A network-centric model for screening and ranking the predicted binding pockets from molecular simulations provides an adaptation and extension of the reversed allosteric communication strategy. By using this model, we discover commonalties along with significant variant-specific differences in the distribution of binding sites in the BA.1 and BA.2 trimers, suggesting that small variations could lead to different preferences in the allocation of druggable sites. We show that the proposed approach can recover the experimentally known allosteric sites in the NTD, RBD, and targetable S2 regions as highly ranked top binding pockets. The results of this study indicate that while both closed and open S-BA.1 trimers featured as highly ranked in the NTD supersite region, the conformational dynamics of the S-BA.2 trimers could mask the NTD’s cryptic region and present previously underappreciated cryptic binding pockets in the inter-protomer interface and hinge regions of the S2 subunit. Using this approach, we demonstrate that clusters of allosteric hotspots which mediate long-range communications in the S trimers could anchor druggable cryptic binding pockets, including the experimental allosteric sites. The results of this study are particularly significant for understanding the universe of cryptic binding sites and variant-specific preferences for druggable pockets. This may have implications for the discovery and design of fusion inhibitors and modulators which, due to their small size and specific conformations, are more likely to bind the cryptic epitopes that are usually inaccessible to conventional antibodies, inducing a more potent cross-neutralizing activity.

## 2. Materials and Methods

### 2.1. Structural Modeling and Refinement

The crystal structures of the S trimers in open and closed forms for the BA.1 and BA.2 variants (Table 1 and Table 2) were obtained from the Protein Data Bank [97]. The following Omicron BA.1 structures were used in our study: the cryo-EM structure of the S Omicron BA.1 3-RBD down trimer without the P986-P987 stabilizing mutations (S-GSAS-Omicron) (pdb id: 7TF8, 3.4 Å resolution) [27]), the S Omicron BA.1 3-RBD down trimer without the P986-P987 stabilizing mutations (S-GSAS-Omicron) (pdb id: 7TL1, 3.5 Å resolution) [27]), the S Omicron BA.1 1-RBD up trimer without the P986-P987 stabilizing mutations (S-GSAS-Omicron) (pdb id: 7TEI, 3.4 Å resolution) [27]), the S Omicron BA.1 1-RBD up trimer (pdb id: 7TL9, 3.5 Å resolution) [27]), the S Omicron BA.1 2-RBD up trimer without the P986-P987 stabilizing mutations (S-GSAS-Omicron) (pdb id: 7TGE 3.7 Å resolution) [27]), the S Omicron BA.1 3-RBD down trimer (pdb id: 7TNW, 3.1 Å resolution) [37]), the S Omicron BA.1 1-RBD up trimer (pdb id: 7TO4, 3.4 resolution) [37]), the S Omicron BA.1 3-RBD down trimer (pdb id: 7WK2, 3.1 Å resolution) [24], the S Omicron BA.1 1-RBD up trimer (pdb id: 7WK3, 3.4 Å resolution) [24], the S Omicron BA.1 1-RBD up trimer (pdb id: 7WVN, 4.0 Å resolution) [24], and the S Omicron BA.1 1-RBD up trimer (pdb id: 7WVO, 4.0 Å resolution) [24]. The following Omicron BA.2 structures were used in this study: the S Omicron BA.2 3-RBD down trimer (pdb id: 7XIX, 3.25 Å resolution) [38], the S Omicron BA.2 1-RBD up trimer (pdb id: 7XIW, 3.62 Å resolution) [38], the S Omicron BA.2 3-RBD down trimer (pdb id: 7UB0, 3.31 Å resolution) [31], the S Omicron BA.2 3-RBD down trimer (pdb id: 7UB5, 3.35 Å resolution) [31], the S Omicron BA.2 3-RBD down trimer (pdb id: 7UB6, 3.52 Å resolution) [31], the S Omicron BA.2 3-RBD down trimer (pdb id: 8D55, 2.8 Å resolution) [37], and the S Omicron BA.2 1-RBD up trimer (pdb id: 8D56, 3.0 Å resolution) [37]. During the structure preparation stage, protein residues in the cryo-EM and crystal structures (Table 2) were inspected for missing residues and protons. Hydrogen atoms and missing residues were initially added and assigned according to the WHATIF program web interface [98]. The protonation states for all the titratable residues of the ACE2 and RBD proteins were predicted at a pH of 7.0, using Propka 3.1 software and a web server [99,100]. The missing segments in the studied structures of the SARS-CoV-2 S protein were reconstructed and optimized using a template-based loop prediction approach, ArchPRED [101]. The side-chain rotamers were refined and optimized using the SCWRL4 tool [102]. The protein structures were then optimized using atomic-level energy minimization with composite physics and knowledge-based force fields, implemented via the 3Drefine method [103]. The atomistic structures from the simulation trajectories were further elaborated via the addition of N-acetyl glycosamine (NAG) glycan residues and then optimized.

In addition to the experimentally resolved glycan residues present in the cryo-EM structures of the studied S proteins, the glycosylated microenvironment was mimicked by using the structurally resolved glycan conformations for 16 out of the 22 most occupied N-glycans (N122, N165, N234, N282, N331, N343, N603, N616, N657, N709, N717, N801, N1074, N1098, N1134, and N1158), as determined in the cryo-EM structures of the SARS-CoV-2 spike S trimer in the closed state (pdb id: 6VXX) and open state (pdb id: 6VYB) and the cryo-EM structure SARS-CoV-2 spike trimer (K986P/V987P) in the open state (pdb id: 6VSB).

### 2.2. Coarse-Grained Dynamics Simulations

Coarse-grained Brownian dynamics (BD) simulations were conducted using the ProPHet (Probing Protein Heterogeneity) approach and program [104]. BD simulations are based on high-resolution CG protein representations [105] of the SARS-CoV-2 S Omicron trimer structures that can distinguish different residues. In this model, each amino acid is represented by one pseudo-atom at the Cα position and two pseudo-atoms for large residues. The interactions between the pseudo-atoms are treated according to the standard elastic network model (ENM) in which the pseudo-atoms within a cut-off parameter, *R*_c_ = 9 Å, are joined by Gaussian springs with identical spring constants of *γ* = 0.42 N m^−1^ (0.6 kcal mol^−1^ Å^−2^). The simulations use an implicit solvent representation via diffusion and random displacement terms and hydrodynamic interactions through the diffusion tensor, using the Ermak–McCammon equation of motions and hydrodynamic interactions, as described in the original pioneering studies that introduced Brownian dynamics for the simulation of proteins [106,107]. The stability of the SARS-CoV-2 S Omicron trimers was monitored in multiple simulations with different time steps and running times. We adopted Δ*t* = 5 fs as a time step for the simulations and performed 100 independent BD simulations for each system, using 500,000 BD steps at a temperature of 300 K. The CG-BD conformational ensembles were also subjected to all-atom reconstruction using the PULCHRA method [108] and CG2AA tool [109] to produce atomistic models of the simulation trajectories.

### 2.3. All-Atom Molecular Dynamics Simulations

In NAMD, the 2.13-multicore-CUDA package [110] with the CHARMM36 force field [111] was employed to perform 500 ns all-atom MD simulations for representative closed and open S-BA.1 structures (pdb id: 7WK2 and 7WK3) [24] and closed and open S-BA.2 trimer structures, respectively (pdb id: 7XIX and 7XIW) [38]. Hydrogen atoms were modeled onto the structures prior to solvation with TIP3P water molecules [112] in a periodic box that extended 10 Å beyond any protein atom in the system. To neutralize the biological system before the simulation, Na^+^ and Cl^−^ ions were added in physiological concentrations to achieve charge neutrality, and a salt concentration of 150 mM of NaCl was used to mimic a physiological concentration. All Na^+^ and Cl^−^ ions were placed at least 8 Å away from any protein atoms and from each other. MD simulations are typically performed in an aqueous environment in which the number of ions remains fixed for the duration of the simulation, with a minimally neutralizing ion environment or salt pairs to match the macroscopic salt concentration [113]. All protein systems were subjected to a minimization protocol consisting of two stages. First, minimization was performed for 100,000 steps with all the hydrogen-containing bonds constrained and the protein atoms fixed. In the second stage, minimization was performed for 50,000 steps with all the protein backbone atoms fixed and for an additional 10,000 steps with no fixed atoms. After minimization, the protein systems were equilibrated in steps by gradually increasing the system temperature in steps of 20 K, increasing from 10 K to 310 K, and at each step, a 1ns equilibration was performed, maintaining a restraint of 10 Kcal mol^−1^ Å^−2^ on the protein C_α_ atoms. After the restraints on the protein atoms were removed, the system was equilibrated for an additional 10 ns. Long-range, non-bonded van der Waals interactions were computed using an atom-based cutoff of 12 Å, with the switching function beginning at 10 Å and reaching zero at 14 Å. The SHAKE method was used to constrain all the bonds associated with hydrogen atoms. The simulations were run using a leap-frog integrator with a 2 fs integration time step. The ShakeH algorithm in NAMD was applied for the water molecule constraints. The long-range electrostatic interactions were calculated using the particle mesh Ewald method [114] with a cut-off of 1.0 nm and a fourth-order (cubic) interpolation. The simulations were performed under an NPT ensemble with a Langevin thermostat and a Nosé–Hoover Langevin piston at 310 K and 1 atm. The damping coefficient (gamma) of the Langevin thermostat was 1/ps. In NAMD, the Nosé–Hoover Langevin piston method is a combination of the Nosé–Hoover constant pressure method [115] and piston fluctuation control implemented using Langevin dynamics [116,117]. An NPT production simulation was run on equilibrated structures for 500 ns, keeping the temperature at 300 K and a constant pressure (1 atm).

### 2.4. Network Analysis of Conformational Ensembles

A graph-based representation of protein structures [118] was used to represent residues as network nodes, and the *inter-residue edges* were used to describe non-covalent residue interactions. The weights of the network edges in the residue interaction networks were determined using dynamic residue cross-correlations obtained from MD simulations [119] and coevolutionary couplings between residues measured using the mutual information scores [120]. The Residue Interaction Network Generator (RING) program [121] was employed for the generation of the initial residue interaction networks. The edge lengths in the network were then adjusted using the generalized correlation coefficients associated with the dynamic correlation and mutual information shared by each pair of residues. The network edges were weighted for residue pairs within at least one independent simulation. A network analysis was performed using the python package NetworkX [122].

The shortest path betweenness of residue *i* was defined as the sum of the fractions of the shortest paths between all pairs of residues that pass through the residue *i*:

(1) $$C_{b}(n_{i})=\sum_{j<k}^{N}\frac{g_{jk}(i)}{g_{jk}}$$

where $g_{jk}$ denotes the number of shortest geodesics paths connecting *j* and *k*, and $g_{jk}(i)$ is the number of shortest paths between residues *j* and *k* passing through the node $n_{i}$. Residues with high occurrences in the shortest paths connecting all residue pairs have higher betweenness values. For each node *n*, the betweenness value was normalized using the number of node pairs excluding *n*, given as (N−1)(N−2)/2, where *N* is the total number of nodes in the connected component that the node *n* belongs to.

The Girvan–Newman algorithm [123] was used to identify local communities. An improvement of the Girvan–Newman method was implemented in which all the highest betweenness edges were removed at each step of the protocol. The algorithmic details of this modified scheme were presented in our recent study [124]. A community-based model of allosteric communications was based on the notion that groups of residues that form local interacting communities are expected to be highly correlated and can switch their conformational states cooperatively. In this model, long-range allosteric communications can be transmitted not between individual residue nodes but rather through a hierarchical chain of local communities connected via inter-modular bridges on the dynamic interaction networks. Using local community decomposition in the protein structures, we identified a hierarchy of clusters consisting of dynamically and coevolutionary coupled nodes (residues at the hierarchical level 1 and meta-nodes formed by small clusters at the next hierarchical level 2). To determine inter-modular bridges between local communities, we employed the inter-community centrality metric [125]. This parameter uses community detection as an input:

(2) $$G\left(i\right)=\sum_{j\in J}l_{IJ}\delta_{iJ}\;$$

where the sum is taken over the community *J* (different from the community of node *i*, which is denoted as *I*); $\delta_{iJ}$ is equal to 1 if there is a link between the node *i* and the community *J* and is 0 otherwise. $l_{iJ}$ corresponds to the distance between community *I* and the community *J*. It is measured using the inverse of the number of links between them. The community centrality is determined according to the following expression:

(3) $$C_{c}\left(i\right)=\sum_{i\in j}^{N}\;\left(\;1-\frac{1}{m}\sum_{i\in j\cap k}^{m}S(j,k)\right)$$

where *N* is the number of communities to which node *i* belongs, and *S* the Jaccard similarity coefficient between the communities *j* and *k*, calculated for the number of shared nodes between each community pair. The sum is averaged over the m communities that are paired with community *j* to which a given node *i* may simultaneously belong. All parameters were computed using the python package NetworkX [122].

### 2.5. Machine Learning-Based Identification of Cryptic Pockets and Network-Based Ranking of Allosteric Pocket Propensities and Allosteric Binding Sites

We used two different complementary approaches for enumerating the putative binding pockets in the conformational ensembles of the S-BA1 and S-BA.2 structures. A template-free P2Rank approach is among the most efficient and fast available ML tools for the prediction of ligand binding sites that combines sequence and structural data to rank potential binding sites based on their likelihood of binding a specific ligand [74,75]. P2Rank uses a support vector machine (SVM), random forests (RFs), and artificial neural networks (ANNs) to learn the ligandability of a local chemical environment that is centered on points placed on a protein’s solvent-accessible surface. P2Rank v2.4 was deployed with its default parameters to identify pockets across all of the representative states from our simulations. P2Rank’s approach predicts ligandability scores using a random forest classifier and clusters points with high ligandability scores to form pocket predictions in which single-linkage clustering with a cut-off of 3Å is used. Finally, P2Rank ranks the predicted pockets using the cumulative ligandability score of their points [74,75].

By combining eXtreme gradient boosting (XGBoost) and graph convolutional neural networks (GCNNs), a robust approach for the identification of allosteric sites and the Prediction of Allosteric Sites Server (PASSer) was developed [77,78,79]. We also employed the PASSer Learning to Rank (LTR) model, which is capable of ranking pockets in order of their likelihood of being an allosteric site. In this approach, FPocket was applied to each protein to detect protein pockets [126]. For each detected pocket, 19 physical and chemical features are calculated, ranging from pocket volume and solvent-accessible surface area to hydrophobicity. The LTR model uses LightGBM [127], one of the two popular implementations of the gradient-boosted decision trees (GBDT) method and XGBoost [128]. Using the P2Rank [73,74] and PASSer LTR [79] approaches, we identified binding pockets in the conformational ensembles and computed the P2Rank-predicted residue pocket probabilities. In the next step, we reweighted the pocket propensity scores and re-ranked the predicted binding pockets, using network-based hierarchical community centralities determined for each protein residue. We then characterized the difference in each residue’s maximum ligand-binding-pocket probability in the ensemble and network-weighted pocket propensities. We employed these methods for the exhaustive detection of possible binding pockets in the conformational ensembles of the studied S proteins. The pockets that maintained their structural composition and were ranked among the top 10 consensus score pockets using both the P2Rank and LTR methods were referred to putative cryptic pockets if one of the following conditions was satisfied: (a) these pockets were not detected in the static structures of the S protein; or (b) the composition of these pockets is appreciably different in the static structures, with >50% different residues comprising the site; or (c) the predicted pocket is predicted at the lower 10% of the pocket score of the static structures. Using these criteria, we considered some of the predicted cryptic sites as pockets that are stable and highly probable in the conformational ensemble while being either completely absent or marginally probable in the static structures and would therefore never have been considered as viable pockets based on a single structure evaluation. In the final step, the predicted cryptic pockets were analyzed using their networking properties, which were estimated based on the cumulative network centrality scores of the comprising residues. The reported pockets were formed by residues with high network centrality scores that mediate allosteric interactions in protein structures. We refer to these cryptic binding pockets as potential allosteric binding sites.

## 3. Results

### 3.1. Conformational Landscapes of Multiple Conformational States of the SARS-CoV-2 S BA.1 and BA.2 Trimers

**Figure 1:**
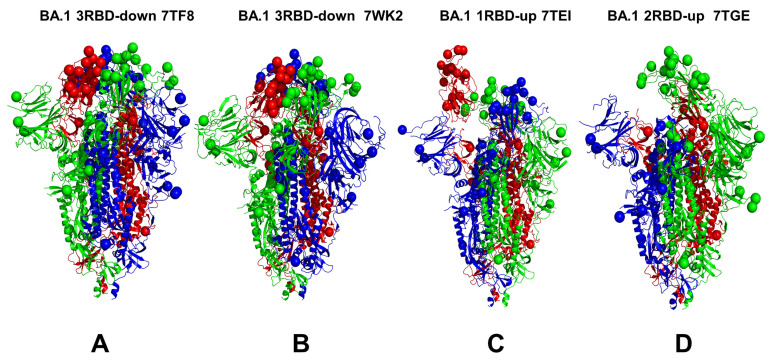
Structural organization and mapping of Omicron BA.1 mutations in SARS-CoV-2 S closed Omicron BA.1 trimers. A general overview of the SARS-CoV-2 S Omicron BA.1 trimer in the 3RBD-down closed form, pdb id 7TF8 (**A**); the BA.1 closed trimer, pdb id 7WK2 (**B**); the BA.1 1RBD-up open trimer, pdb id 7TEI (**C**); and the BA.1 2RBD-up open trimer, pdb id 7TGE (**D**). Protomer A is shown in green ribbons, protomer B is shown in red ribbons, and protomer C is shown in blue ribbons. The positions of the Omicron mutations are shown for each protomer in spheres colored according to the respective protomer.

**Figure 2:**
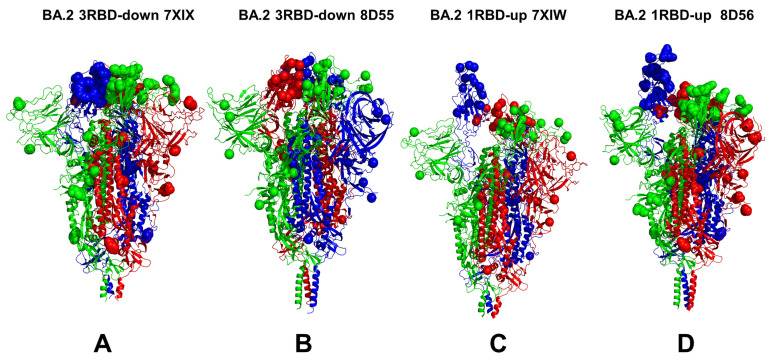
Structural organization and mapping the Omicron BA.2 mutations in SARS-CoV-2 S closed Omicron BA.2 trimers. A general overview of the SARS-CoV-2 S Omicron BA.2 trimer in the 3RBD-down closed form, pdb id 7XIX (**A**); the BA.2 closed trimer, pdb id 8D55 (**B**); the BA.2 1RBD-up open trimer, pdb id 7XIW (**C**); and the BA.2 1RBD-up open trimer, pdb id 8D56 (**D**). Protomer A is shown in green ribbons, protomer B is shown in red ribbons, and protomer C is shown in blue ribbons. The positions of the Omicron BA.2 mutations The Omicron BA.2 mutational sites are shown for each protomer in spheres colored according to the respective protomer.

We combined multiple coarse-grained simulations followed by atomistic reconstruction of the trajectories to perform a comparative analysis of the conformational landscapes of the S Omicron BA.1 (Figure 1) and BA.2 trimers (Figure 2). A total of 12 Omicron mutations (G339D, S373P, S375F, K417N, N440K, S477N, T478K, E484A, Q493R, Q498R, N501Y, and Y505H) are shared among the BA.1 and BA.2 variants. In the RBD, BA.1 contains the unique mutations S371L, G446S, and G496S while BA.2 carries the S371F, T376A, D405N, and R408S mutations (Table 1). The BA.1 and BA.2 S trimers are different in three additional residues: T547K in the S1 region and D856K and L981F in the S2 region, which are found in the BA.1 variant but not in the BA.2 variant (Table 1). In the conformational dynamics analysis, we simulated the cryo-EM structures of the unbound S-BA.1 and S-BA.2 trimers in the 3 RBD-down closed form, various 1RBD-up open conformations, and the 2RBD-up state (Table 2). Coarse-grained Brownian dynamics (CG-BD) was employed within the ProPHet approach [104] that combines an elastic network (ENM) protein model and a Brownian Dynamics algorithm to compute local protein rigidity on the residue level [129,130]. We performed 100 independent simulations for each of the studied S-BA.1 and S-BA.2 trimer structures (Table 2). In addition, atomistic MD simulations were conducted for representative closed and open S-BA.1 trimer conformations (pdb id: 7WK2 and 7WK3), as well as closed and open S-BA.2 trimer structures (pdb id: 7XIX,7XIW) (Table 3). The use of multiple S Omicron trimer structures in the CG-BD and MD simulations (Table 2 and Table 3) allowed for a comparative analysis of protein dynamics in the distinct S Omicron states.

**Figure 3:**
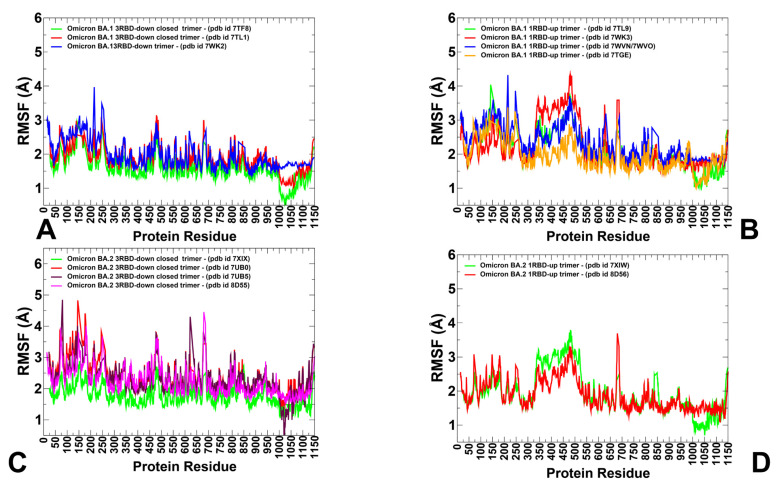
Conformational dynamics profiles obtained from multiple CG-BD and MD simulations of the Omicron BA.1 and BA.2 trimers. (**A**) The RMSF profiles for the S protein residues obtained from simulations of the S-BA.1 closed trimers (pdb id 7TF8 in green lines, pdb id 7TL1 in red lines, and pdb id 7WK2 in blue lines). (**B**) The RMSF profiles for the S protein residues obtained from simulations of the S-BA.1 1 RBD-up open trimers (pdb id 7TL9 in green lines, pdb id 7WK3 in red lines, pdb id 7WVN/7WVO in blue lines), and S-BA.1 2RBD-up open trimer (pdb id 7TGE in orange lines). (**C**) The RMSF profiles for the S protein residues obtained from simulations of the S-BA.2 closed trimers (pdb id 7XIX in green lines, pdb id 7UB0 in red lines, pdb id 7UB5 in maroon lines, and pdb id 8D55 in magenta lines). (**D**) The RMSF profiles for the S protein residues obtained from simulations of the S-BA.2 1 RBD-up open trimers (pdb id 7XIW in green lines and pdb id 8D56 in red lines). The RMSF profiles represent the results of averaging over multiple CG-BD runs.

A conformational dynamics analysis of the S-BA.1 and S-BA.2 closed conformations showed generally similar root-mean-square fluctuation (RMSF) profiles, reflecting the stability of the 3-RBD structure for both variants (Figure 3A,B). The profiles showed similar thermal fluctuations in the RBD-closed S1 subunit (residues 14–530) and the more rigid S2 subunit (Figure 3A,B). Greater deviations were detected in the NTD residues 14–306, but the pattern of thermal fluctuations in this region differed between the BA.1 and BA.2 trimers. In the S-BA.1 trimer, we observed larger RMSF displacements in the NTD residues 75–250 (Figure 2A), while for the S-BA.2 variant, we observed several NTD peaks corresponding to the residues 60–80, 140–180, and 240–260 (Figure 3B). In the Omicron BA.1 and BA.2 closed trimers, the upstream helix (UH) (residues 736–781) and central helix (CH) (residues 986–1035) are particularly rigid, while the C-terminal domain 1 (CTD1) (residues 528–591) and C-terminal domain 2 (CTD2) (residues 592–686) undergo moderate fluctuations (Figure 3A,B). For both variants, thermal fluctuations were observed in the RBD residues 466–498 and the intrinsically flexible CTD2 region (residues 615–640), as well as in the furin cleavage region (residues 670–690) (Figure 3). The S1/S2 cleavage site lies on an exposed flexible loop and is often missing in cryo-EM structures due to its high mobility. In both the S-BA.1 and S-BA.2 trimers, P681H mutations near the furin cleavage site (residues 682–685) may increase S1/S2 cleavage as this mutation enhances the exposure of this site, confers greater structural flexibility to this region, and probably decreases the thermostability of proteins. The observed RMSF fluctuations in this region are consistent with the experimental data and B-factors in the cryo-EM structures (Table 2), reflective of the intrinsic flexibility and plasticity of this region. The proteolytic cleavage of the S protein at its S1/S2 and S2 junctions is extensively used to trigger the fusion apparatus of viral glycoproteins in which the S2 subunit of S promotes the membrane-fusion step of viral entry by undergoing dynamic conformational changes. Consistent with strong sequence conservation, the CH region is exceedingly rigid in the S-BA.1 and S-BA.2 closed trimers. Interestingly, the conformational dynamics profiles showed signs of greater rigidity in the S2 subunit but more flexibility in the S1 subunit (particularly in the NTD) for the S-BA.1 closed trimer (Figure 3A). At the same time, the RMSF profiles indicated moderately greater degrees of plasticity of both the S1 and S2 subunits for the BA.2 variant (Figure 3B). The inter-protomer salt bridges K856-D571 in the BA.1 timers and the absence of these stabilizing interactions in the BA.2 trimer may contribute to some plasticity in the S1-S2 and S2 regions of the S-BA.2 trimer.

However, the differences in the RMSF profiles of the closed trimers for the BA.1 and BA.2 variants remained moderate, suggesting the thermal stability of the closed states and the only marginally increased mobility of the S-BA.2 trimers. A comparison of the conformational dynamics profiles obtained from the simulations of the RBD-up open states for the S-BA.1 and S-BA.2 trimers revealed several interesting patterns (Figure 3C,D). It was observed that the RMSF distributions for the open S-BA.1 structures showed a larger degree of heterogeneity and greater fluctuations in the NTD and exposed RBD-up regions (Figure 3C). In some contrast, the conformational flexibility of the NTD and RBD in the open BA.2 trimers was moderately reduced, displaying smaller displacements within an RMSF value < 3 Å (Figure 3D). Based on these fundings, we argue that the RBD-up conformations for the S-BA.1 trimer may experience greater variability in the S1 subunit while the open states for the S-BA.2 trimer become more stable, thus suggesting that the open S form could become more dominant in the BA.2 variant. These findings are consistent with the experiments showing the increased stability of the RBD and the enhanced propensity to populate open trimer states of the BA.2 variant [36].

In addition to the variability of the RBD-up regions, we observed differences in the mobility of the NTD. In this context, it is worth noting that the NTD region (residues 14–20 and the NTD N-terminus), N3 (residues 141–156), and N5 (residues 246–260) collectively form an antigenic supersite on the NTD. These regions showed increased RMSF values for both variants, but the degree of conformational heterogeneity appeared to be greater in the S-BA.2 conformations (Figure 3). This suggests that conformationally flexible NTD regions in the S-BA.1 trimers may preserve the topology of the NTD supersite, while specific NTD mutations and the increased heterogeneity of the NTD regions in the BA.2 variant could promote the reconstruction of the antibody NTD site and lead to the formation of dynamic pockets on the surfaces of the NTD. These dynamic characteristics gleaned from the RMSF analysis are consistent with the results of thermal stability assays that verified that S BA.2 trimers are the least stable among the BA.1, BA.2, BA.3, and BA.4 variants [39].

While the analysis of the MD simulations provided important insights into the underlying conformational landscapes of the BA.1 and BA.2 trimers, the high dimensionality of data sets produced via simulations often hinders salient dynamic differences between Omicron variants. To better understand key differences between the conformational landscapes of the BA.1 and BA.2 trimers and to further examine the Omicron-induced modulation of S dynamics, we also performed a principal component analysis (PCA). Using this approach, we were able to reduce a multidimensional, complex set of variables to a lower dimension and extract information from the sampled conformations over a trajectory. A PCA is used to systematically reduce the number of dimensions needed to describe protein dynamics through a decomposition process that filters observed motions from the largest to smallest spatial scales [131,132]. The low-dimensionality projection of the MD ensemble in different microsecond trajectories highlighted a consistent occupancy of the dominant conformational region for the BA.2 complex pocket (Supplementary Materials, Figure S1). In the PCA, we combined CG-BD simulation trajectories and projected multidimensional data onto a two-dimensional PCA plot for each of the studied S-BA.1 trimers and S-BA.2 trimers (Supplementary Materials, Figure S1). By combining multiple conformational ensembles together, we were able to increase statistics and better characterize differences in the dynamic landscapes of the BA.1 and BA.2 trimers. The combined conformational ensembles of the closed and open states for each of the Omicron variants allowed for the identification of local basins and the paths connecting them (Supplementary Materials, Figure S1). Interestingly, we observed both expected similarities arising from very similar structural organizations of the trimer folds as well as notable differences reflecting a more dynamic and adaptable nature of the BA.2 trimer’s closed conformations. It can be seen that for S-BA.1 trimers, the results of the PCA point to dense area associated with closed conformations and a somewhat more diffused local basin of open conformations (Supplementary Materials, Figure S1A). At the same time, for the S-BA.2 trimers, we observed a broadening of the distribution (Supplementary Materials, Figure S1B), suggesting the larger conformational space and increased variability of the BA.2 variant. The overlap between the PCA results for the BA.1 and BA.2 trimers indicates important similarities, especially for the closed conformations, while showing that BA.2 can explore more conformational space (Supplementary Materials, Figure S1C), which may be indicative of this variant’s greater functional adaptability.

### 3.2. The Functional Dynamics of the BA.1 and BA.2 Trimers Highlight the Complementary Roles of the Omicron Mutational Sites as Hinges and Transmitters of Collective Motions

**Figure 4:**
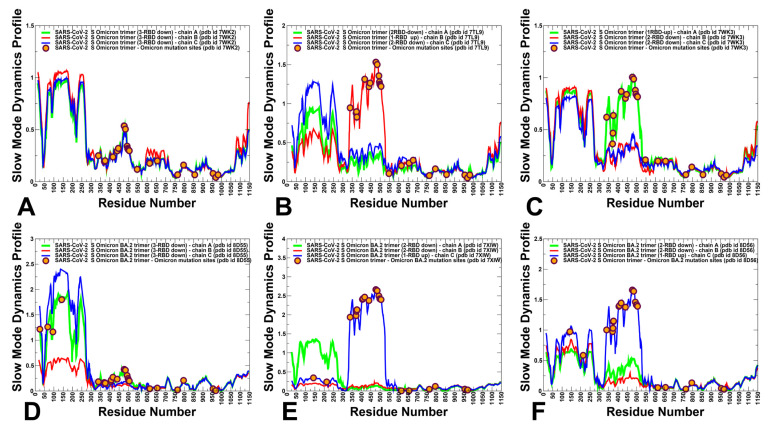
The slow-mode mobility profiles of the SARS-CoV-2 S Omicron trimer structures. The slow-mode dynamics profiles represent the displacements along slow-mode eigenvectors and correspond to the cumulative contribution of the slowest five modes. The slow-mode mobility profiles for the following S-BA.1 trimer states are shown: the 3RBD-down closed BA.1 trimer, pdb id 7WK2 (**A**); the 1RBD-up open BA.1 trimer, pdb id 7TL9 (**B**); and the 1RBD-up open BA.1 trimer, pdb id 7WK3 (**C**). The slow-mode mobility profiles for the following S-BA.2 trimer states are shown: the 3RBD-down closed BA.2 trimer, pdb id 8D55 (**D**); the 1RBD-up open BA.2 trimer, pdb id 7XIW (**E**); and the 1RBD-up open BA.2 trimer, pdb id: 8D56 (**F**). The slow-mode profiles for protomer chains A, B, and C are shown in green, red, and blue lines, respectively. The positions of the Omicron BA.1 and BA.2 mutational sites are shown in filled, orange-colored circles. The results were obtained via averaging over multiple simulation runs.

To characterize collective motions in the SARS-CoV-2 S Omicron structures, we determined the five low-frequency modes and slow-mode displacement profiles which correspond to the reported cumulative contribution of these modes (Figure 4). The slow-mode profiles can detect key mechanical hinge sites that can control collective motions and also characterize regions susceptible to global structural transformations. The local minima along the slow-mode profiles are typically aligned with the hinge centers, while the maxima could point to moving regions undergoing concerted movements. The slow-mode shapes revealed important similarities indicating the conservation of major hinge centers. The key functional signatures of collective dynamics in these states are the large displacements of the NTD and RBD regions that enable the S protein to assume a receptor-accessible conformation (Figure 4). The important finding of this analysis is the correspondence of the major hinge positions with the Omicron sites in the S2 subunit (Figure 4). The slow-mode profiles in the closed S-BA.1 states showed that the residues N764K, N856K, Q954H, N969K, and L981F corresponded to the local minima of the distribution (Figure 4A–C). Interestingly, the key immobilized hinge positions were conserved and shared by both the closed and open forms of the S-BA.1 and S-BA.2 trimers.

A comparison of the slow modes for the closed forms of the S-BA.1 (Figure 4A) and S-BA.2 trimers (Figure 4D) indicated interesting differences. In the S-BA.1 closed trimer, the closed-down RBDs experience moderate movements in collective motions, while the NTD regions may experience functional displacements (Figure 4A). As may be expected, in the open BA.1 conformations, we observed significant functional displacements of the RBD region, indicating that the Omicron mutations’ target sites are involved in collective movements (Figure 4B,C). Consistent with our previous studies of the S trimer protein, the functional dynamics profiles also revealed that the F318, A570, I572, and F592 residues are conserved hinge sites that are situated near the inter-domain CTD1-S2 interfaces and could function as regulatory centers governing functional transitions (Figure 4B,C). A comparison of the slowest five modes for the S-BA.1 closed trimer showed the dominant contribution of the NTD movements and appreciable displacements occurring in the RBD and CTD1 regions, with the S2 regions remaining near their native positions (Supplementary Materials, Figure S2A). For the BA.1 1RBD-up trimers, the top mode was associated with the functional RBD movements, while the remaining slowest modes displayed concerted motions of both the NTD and RBD regions (Supplementary Materials, Figure S2B,C). Only very small deviations along the slowest five global modes were seen in the S2 regions of the BA.1 structures (Supplementary Materials, Figure S2A–C), suggesting that the structurally rigid S2 subunit is largely immobilized in global motions and serves as an anchoring unit to enable the diverse displacements of NTD, RBD and CTD1 regions.

Our analysis showed that the functional movements of the NTD regions can become more pronounced and diverse among the three protomers in the S-BA.2 closed trimer (Figure 4D). These observations suggest a greater heterogeneity of the functional movements in the BA.2 closed state. A more detailed inspection of the functional displacements along each of the five slowest modes revealed interesting and unexpected findings (Supplementary Materials, Figure S2D,E). In particular, for the closed BA.2 structures, even though the slowest mode was associated primarily with the movements of the NTD, other slow modes showed appreciable deviations not only for the S1 regions (RBD and CTD1) but also for the S2 regions (residues 750–800 and 850–950) (Supplementary Materials, Figure S2D). Some of these displacements appeared to include the HR1 region (residues 910–985), indicating the increased plasticity of the S2 subunit and particularly the HR1 region. Recent HDX-MS studies showed that the allosteric binding of ACE2 perturbs and primes HR1 and the regions flanking the S1/S2 cleavage site for cleavage [133,134,135].

There were also differences between the slow-mode profiles for the open trimer states of the BA.1 and BA.2 variants. For the S-BA.1 open trimer, the profiles showed significant displacements of both the NTD and RBD regions (Figure 4B,C). Moreover, functional NTD motions can occur in both the closed and open protomers of the 1RBD-up BA.1 trimer, leading to significant variations in these regions, while the S2 subunit is immobilized. The S-BA.1 trimers may be prone to conformational transformations between open and closed forms in the absence of a host receptor such that ACE2 binding promotes a shift to the open form. For the open states of S-BA.2, the movements of the S1 subunit become mainly associated with the RBD’s movements, while the NTD’s displacements are less significant (Figure 4E,F). This finding is further supported by a more detailed inspection of the slow-mode profiles for each of the slowest modes for which we clearly observed the dominant contribution of the RBD’s motions and smaller displacements in the NTD regions (Supplementary Materials, Figure S2E,F). This analysis supports a mechanism in which the increased plasticity of the conformation of the S-BA.2 trimers may lead to a lowered barrier between the closed and open forms and facilitate the acquisition of adaptable 1RBD-up states. This may cause the higher transmission and infection rates of the BA.2 Omicron sublineage compared to the BA.1 variant.

Conformational dynamics profiles corresponding to the cumulative contributions of the five slowest modes were projected onto S trimer structures (Supplementary Materials, Figure S3). The structural maps showed the stability of the S2 regions and the considerable functional mobility of both the NTD and RBD regions in functional motions. This analysis also highlighted the importance of the inter-domain SD1-S2 interfaces that connect the immobilized and more flexible units of the S trimer, thus presenting functionally important hinges that control the relative movements of the S1 regions. Interestingly, while the cumulative contributions of slow modes may be similar in the BA.1 and BA.2 trimers’ structures, an analysis of the functional dynamics along the slowest mode showed some differences (Supplementary Materials, Figure S4). Indeed, we observed some weakening of the structural rigidity in the BA.2 closed trimer (Supplementary Materials, Figure S4D), with the NTD and RBD regions undergoing functional movements. A similarly increased conformational plasticity was also seen in open BA.2 structures in which significant fractions of the S1 and S2 units may experience displacements along the slowest mode (Supplementary Materials, Figure S4E,F). Accordingly, this may indicate the increased conformational heterogeneity of the S-BA.2 trimer’s states and its decreased stability. The observations from the functional dynamics analysis are supported by the findings from thermal shift assays which showed an observed lower temperature for the dissociation of the S-BA.2 trimer [36]. Moreover, this experimental study showed the the dissociation constant of the BA.2 S protein with ACE2 is fivefold and twofold higher than the dissociation constants of the wild-type S protein and S-BA.1 protein with ACE2, respectively [36]. Our analysis of collective movements suggests that the evolution of the Omicron variants may be accompanied by subtle changes in the intrinsic dynamic patterns, leading to a progressive shift toward more a stable open form for the BA.2 variant. The observed patterns of collective motions in the S-BA.1 and S-BA.2 states are also consistent with recent experiments showing the increasing flexibility of the NTD regions in the Omicron trimers which may enable the escape of NTD-targeting antibodies, thereby providing an evolutionary advantage. The observed differences in the modulation of the NTD’s mobility between the BA.1 and BA.2 conformations support the proposed mechanism in which the NTD provides an adaptable antigenic surface, allowing for enhanced immune escape potential [41].

Interestingly, several Omicron sites belong to local hinge regions (N764K, D796Y, N856K, Q954H, N969K, and L981F), while the RBD’s mutational sites are located in regions prone to functional movements. The hinge sites F318, A570, T572, F592, D614G, N764K, N856K, Q954H, and N969K participate in direct interaction contacts that can strengthen the stability of the hinge clusters in S-BA.1 trimers. In particular, N856K interacts with T572, while N764K makes favorable contacts with the T315, N317, and Q314 positions of the adjacent protomer. N969K is hydrogen-bonded with the sites Y756 and Q755, while T547K is in direct contact with the S982 and L981 positions. The Omicron mutations in these regions introduce new stabilizing contacts that promote the structural rigidity and expansion of the hinge clusters. We also noticed that the F318, S383, A570, I572, F592, and D985 residues are conserved hinge sites that are situated near the inter-domain SD1-S2 interface and could function as regulatory centers governing the inter-protomer and inter-domain transitions (Figure 4, Supplementary Materials, Figures S2–S4). In both the closed and open forms of the S Omicron variants, the NTD Omicron mutational sites experience large displacements and allow for the remodeling of the NTD binding epitope, thus reducing the effect of antibody-mediated protection. At the same time, the RBD Omicron sites in the open state are highly mobile in collective dynamics regions (Figure 4, Supplementary Materials, Figure S2A). Given that Omicron mutational positions in the S2 subunit tend to occupy immobilized hinge sites, this suggests that long-range communications and allosteric cross-talk between the S2 and S1 subunits may be orchestrated and modulated by Omicron mutations. In this context, it may be also argued that the increased dynamic preferences of BA.2 for open states with flexible NTD and RBD regions may promote binding with ACE2 while leveraging the adaptability of the NTD’s binding surfaces to escape immunity.

### 3.3. Dynamic Network Analysis: The Variant-Induced Modulation of Allosteric Mediating Centers

**Figure 5:**
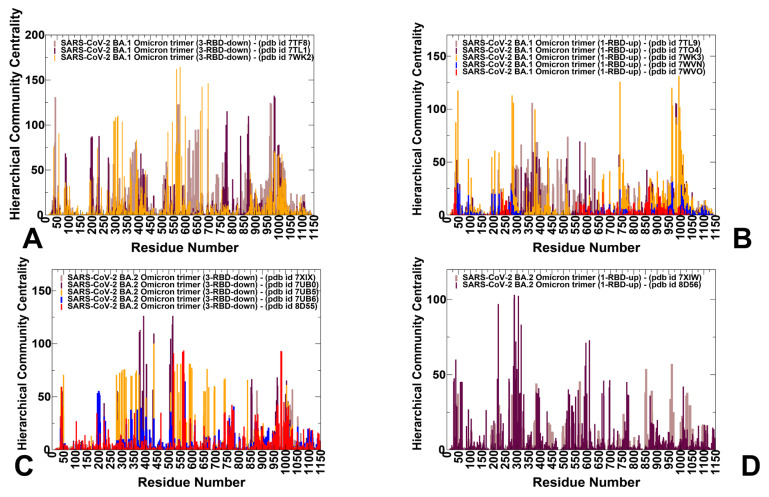
The hierarchical community centrality distributions for the structures of SARS-CoV-2 S Omicron BA.1 and BA.2. (**A**) The centrality profiles for the for the closed S-BA.1 trimers (pdb id 7TF8 in brown, pdb id 7TL1 in maroon, and pdb id 7WK2 in orange-colored bars). (**B**) The centrality profiles for the for the open S-BA.1 trimers (pdb id 7TL9 in brown, pdb id 7TO4 in maroon, pdb id 7WK3 in orange, pdb id 7WVN in magenta, and pdb id 7WVO in red bars). (**C**) The centrality profiles for the for the closed S-BA.2 trimers (pdb id 7XIX in brown, pdb id 7UB0 in maroon, pdb id 7UB5 in orange, pdb id 7UB6 in magenta, and pdb id 8D55 in red bars). (**D**) The centrality profiles for the for the open S-BA.2 trimers (pdb id 7XIW in brown and pdb id 78D56 in maroon bars). The distributions were obtained via averaging computations over conformational ensembles of studied systems.

We used hierarchical network modeling to characterize the organization of residue interaction networks in the S Omicron functional states. We constructed and analyzed the dynamic residue interaction networks using an approach in which the dynamic fluctuations obtained from the simulations were mapped onto a graph, with nodes representing residues and edges representing the weights of the measured dynamic properties. The hierarchical community centrality metric was employed in this network model to identify the S protein positions that mediate allosteric communications between local communities (Figure 5). As a result, residues featuring high community centrality values may function as inter-community connectors. For clarity of presentation and consistency, we report the distributions of community centrality in which the inter-community bridges are represented at the single-residue level. We found that in the BA.1 closed trimers, the distinct maxima of the network distribution corresponded to clusters of residues in the NTD (residues 50–60, 100–120, and 200–207) (Figure 5A). Strikingly, some of these NTD residues contribute to the dynamically evolving binding pocket formed by the residues I101, W104, I119, V126, M177, F192, F194, I203, and L226, which form the interactions with the ligand in the cryo-EM structure [55,56]. These NTD peaks are mostly preserved in the open S-BA.1 trimers (Figure 5B), but the contributions of these regions to the network distribution is modestly reduced.

Importantly, we found that for all the protomers in the closed state, the dominant centrality peak corresponded to the N2R linker region (residues 300–336) that connects the NTD and RBD within a single protomer unit, the CTD1 region, and the strong centrality density in the NTD regions’ positions (Figure 4A,B). In the S-BA.1 trimers, we also noticed a significant peak associated with the RBD-CTD1 interfaces that harbor stable local communities of residues (C-535 C-554 C-583; A-544 A-564 A-579; B-330 B-544 B-579; C-544 C-564 C-579). The important mediating roles of the inter-protomer interfaces are exemplified by the peaks associated with the bridging communities linking neighboring protomers (B-855 C-589 C-592; A-568 A-574 C-854; A-740 A-857 B-592; B-775 B-864 C-665; A-592 C-737 C-855). A broad distribution of high-centrality regions in the closed S-BA.1 trimer corresponds to CTD1 (residues 528–591) and CTD2 (residues 592–686), which link the RBD to the S2 regions (Figure 5A). These peaks are weakened in the open BA.1 trimer, thus highlighting changes in allosteric communications between the closed and open forms (Figure 5B). The maxima of the distribution profiles are associated with the residues involved in the inter-protomer bridges N764K-Q314, S982-T547K, N856K-D568, N856K-T572, N969K-Q755, N969K-Y756, S383-D985, F855-G614, V963-A570, N317-D737, R319-D737, R319-T739, R319-D745, and K386-F981. The dense cluster peaks of the network profiles in the S2 regions correspond to a stable UH region (residues 736–781) and CH region (residues 986–1035) in which the network centrality peaks often align with the hinge regions (residues 569–572, 756–760). A very pronounced centrality peak is aligned with the HR1/HR2 linker in the S2 subunit (residues 990–1050) that connects HR1 (residues 910–985) and HR2 (residues 1163–1206) (Figure 5B). The local communities in the HR1/HR2 region (C-1083 C-1088 C-1137; B-1032 B-1043 B-1048; B-1032 B-1048 B-1051; A-1028 A-1043 A-1064; A-1005 B-1005 C-1005) enable the stabilization of the S2 regions.

We observed some noticeable weakening of the network centrality profiles in the S-BA.2 trimers (Figure 5C,D) which signals the more dynamic and diffuse nature of the interactions for BA.2. The contribution of the NTD regions is reduced compared to the other profile peaks. At the same time, in the closed S-BA.2 trimer, the largest peak is shifted towards the NTD-RBD and RBD regions. as evidenced by the high centrality values for the residues 350–450 and 500–530 of the RBD (Figure 5C). The RBD-CTD1 interface resides and the HR1-HR2 regions also contribute significantly to the distribution. The profile of the open S-BA.2 trimer is less dense, suggesting the increased heterogeneity of the long-range communications while preserving topologically important mediating clusters in the NTD, RBD, and CTD1 regions (Figure 5D). Overall, the analysis confirmed the hypothesis that S-BA.2 trimers may exhibit greater conformational plasticity and dynamic changes, which may suggest more room for opening and closing cryptic binding pockets in the S1 and S2 subunits. We also propose that the distribution of the potential allosteric pockets in the S-BA.2 trimers may be more sensitive to structural variations and dynamic changes and display more a heterogeneous pocket landscape, while the greater structural stability of the S-BA.1 trimers may lead to some alterations in the repertoire of potential binding sites.

### 3.4. Allostery-Guided Network Screening of Cryptic Binding Pockets in the S-BA.1 and S-BA.2 Conformational Ensembles: Variant-Specific Modulations of the NTD Binding Sites

**Figure 6:**
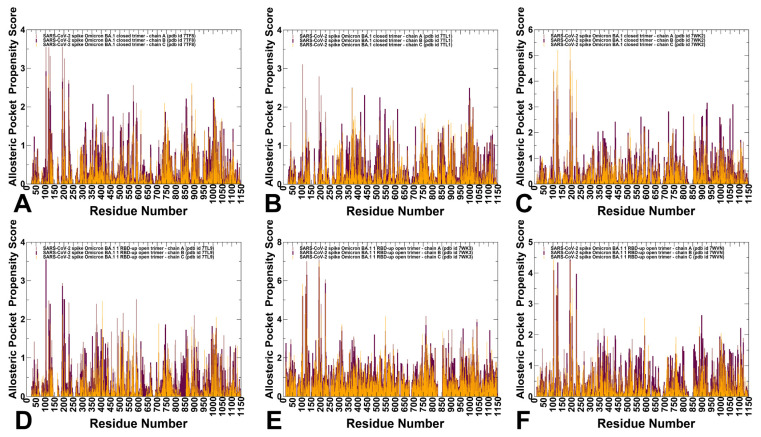
The residue-based pocket propensity distributions for the SARS-CoV-2 S Omicron BA.1 conformations obtained from simulation-based ensembles of structures. (**A**–**C**) The allosteric pocket propensity profile for the closed S-BA.1 trimers (pdb id 7TF8 panel (**A**), pdb id 7TL1 panel (**B**), and pdb id 7WK2 panel (**C**)). (**D**–**F**) The allosteric pocket propensity profile for the open S-BA.1 trimers (pdb id 7TL9 panel (**D**), pdb id 7WK3 panel (**E**), and pdb id 7WVN/7WVO panel (**F**)). The profiles in each structure are shown for the three protomers: protomer A is shown in light-brown bars, protomer B is shown in maroon bars, and protomer C is shown in orange bars.

**Figure 7:**
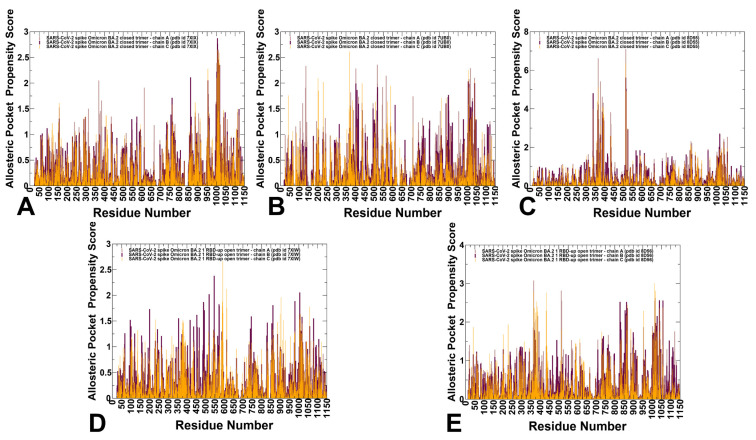
The residue-based pocket propensity distributions for the SARS-CoV-2 S Omicron BA.2 conformations obtained from simulation-based ensembles of structures. (**A**–**C**) The allosteric pocket propensity profile for the closed S-BA.2 trimers (pdb id 7XIX panel (**A**), pdb id 7UB0 panel (**B**), and pdb id 8D55 panel (**C**)). The allosteric pocket propensity profile for the open S-BA.2 trimers (pdb id 7XIW panel (**D**), and pdb id 8D56 panel (**E**). The profiles in each structure are shown for the three protomers: protomer A is shown in light-brown bars, protomer B is shown in maroon bars, and protomer C is shown in orange bars.

We employed conformational ensembles of the S-BA.1 and S-BA.2 trimers in combination with template-free and highly efficient P2Rank and PASSerRank approaches to describe the available spectrum of potential binding sites and compute the residue-based pocket propensities in the ensembles of the S proteins. We combined these robust tools for the enumeration of potential pockets with a network-based weighing of the pocket probabilities to enable pocket ranking based on allosteric function which provides an adaptation and extension of the reversed allosteric communication strategy [81,82,83,84,85,86]. The central result of this analysis is the discovery of variant-specific differences in the distribution of binding sites in the BA.1 and BA.2 trimers, suggesting that small variations could lead to different preferences in the allocation of druggable sites (Figure 6 and Figure 7). First, we determined potential binding pockets in the conformational ensembles and evaluated the ligand-binding-pocket propensities for each protein residue. We propose that by using a network-based, hierarchical community centrality parameter as an allostery-based weighting factor, we can adjust the binding-pocket residue propensities and provide rankings of the binding pockets emerging in the conformational ensembles of the S Omicron trimers. By identifying local residue clusters that correspond to the major peaks of the network centrality distributions, we inferred the positions of regions that could overlap with binding pockets involved in mediating long-range interactions and communications (Figure 6 and Figure 7). In our model, we assumed that the predicted binding pockets that overlap and harbor high-network-centrality sites may correspond to functionally relevant allosteric sites and allow for the effective screening/ranking of the identified pockets. Accordingly, the residue-based binding-pocket probabilities in the ensemble obtained from the P2Rank and PASSerRank calculations were weighted based on the residue network centrality values. The reported pocket propensity scores represent ensemble-averaged values, and pockets that are persistent in the equilibrium trajectories are reported (Figure 6 and Figure 7).

We first analyzed the binding-pocket propensity scores for the closed and open S-BA.1 trimer conformations (Figure 6). The central finding of this analysis is the surprising emergence of the highest distribution peaks aligned with the NTD regions in the closed S-BA.1 trimers (Figure 6A–C). Consistently, the NTD regions (including residues I101, R102, W104, I119, N121, V126, I128, S172, R190, F192, F194, I203, H207, L226, D227, S94, and N99) corresponded to the highest peaks, implying the high propensity of these residues to form an allosteric binding pocket. Moreover, these NTD residues are aligned with high binding-pocket propensities in each of the three closed protomers.

The predicted NTD pocket residues overlap with the experimentally known NTD supersite formed by residues 14–20, residues 140–158 (the supersite b-hairpin), and residues 245–264 (the supersite loop). NTD-neutralizing monoclonal antibodies target the same antigenic supersite, providing examples of convergent solutions to NTD-targeted monoclonal antibody neutralization [57]. The favorable pocket propensity scores for the NTD residues were also found for the 1RBD-up S-BA.1 conformations (Figure 6D–F). Smaller but notable peaks are associated with clusters of residues in the RBD region, residues from CTD1, and local clusters in the S2 subunit corresponding to the HR1 and CH regions (Figure 6A–C). Interestingly, while the distribution of binding-pocket propensities for the S-BA.1 forms is mainly determined by the residues from the dynamic S1 subunit, a significant fraction of the profile are associated with the HR1 and CH regions of the rigid S2 subunit, thereby indicating that an appreciable level of plasticity is present in the S2 regions, giving rise to the broader accumulation of dynamic cryptic pockets.

The BA.1 and BA.2 S proteins differ in the NTD, with only the G142D substitution shared between the two Omicron variants. The S-BA.2 NTD lacks the H69-V70 deletion (ΔH69-V70) that is present in the BA.1 variant, as well as in the Alpha (B.1.1.7) variant. The BA.2 NTD also lacks the deletion of residues 143–145 and the insertion of three residues at position 214. The distribution of network-weighted binding-pocket propensities in the closed S-BA.2 conformations showed a broader distribution of favorable pocket sites in the S1-S2 interfaces and the inter-protomer regions of the S2 subunit (Figure 7A–C). Notably, the high distribution peak corresponding to the NTD regions in the S-BA1 conformations is conspicuously absent for the S-BA.2 ensembles, suggesting that the variant-specific remodeling of the NTD may lead to potential alterations and a redistribution of cryptic sites. In the S-BA.2 conformations, the NTD residues featured moderate binding-pocket propensity values, indicating that the NTD supersite may become partly remodeled and less favorable for ligand binding. These findings may have particular importance in light of the mechanism through which Omicron and other VOCs harbor frequent mutations within the NTD supersite, pointing to antibody-mediated evolutionary pressure to leverage this domain for antibody evasion [136].

At the same time, the largest peaks in the closed and open S-BA.2 conformations are shifted towards S1-S2 inter-domain regions and particularly residue clusters from the HR1 and CH regions of the S2 subunit (Figure 7). These findings signal that Omicron variants may have evolved to increase the conformational plasticity and adaptability of the S2 regions while maintaining functionally significant changes in the NTD and RBD regions. In other words, our results suggested a broadening of residue pocket propensities in S-BA.2 conformations, leading to a more heterogeneous allocation of binding pockets across both the S1 and S2 regions.

### 3.5. Deciphering the Anatomy of Cryptic Binding Pockets in the S-BA.1 and S-BA.2 Conformational Ensembles

**Figure 8:**
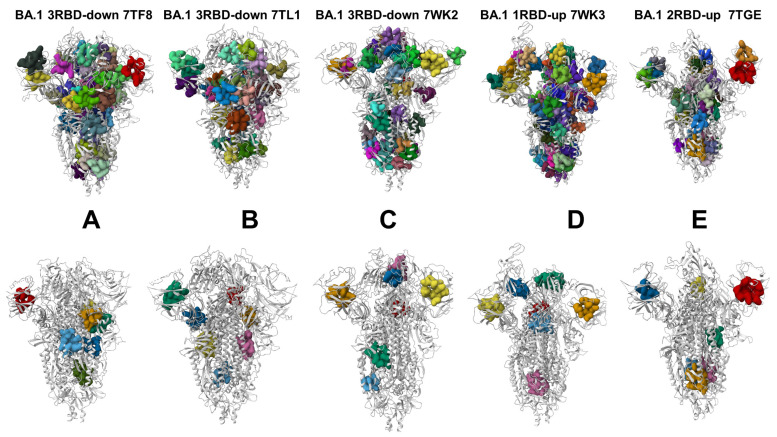
Structural maps of all identified pockets versus the predicted cryptic binding sites for the ensemble of different S-BA.1 structures. Structural map of the predicted pockets for the closed S-BA.1 trimer, pdb id 7TF8 (**A**), closed BA.1 trimer, pdb id 7TL1 (**B**), closed S-BA.1 trimer, pdb id 7WK2 (**C**), open BA.1 trimer, pdb id 7WK3 (**D**), and open BA.1 trimer, pdb id 7TGE (**E**).

**Figure 9:**
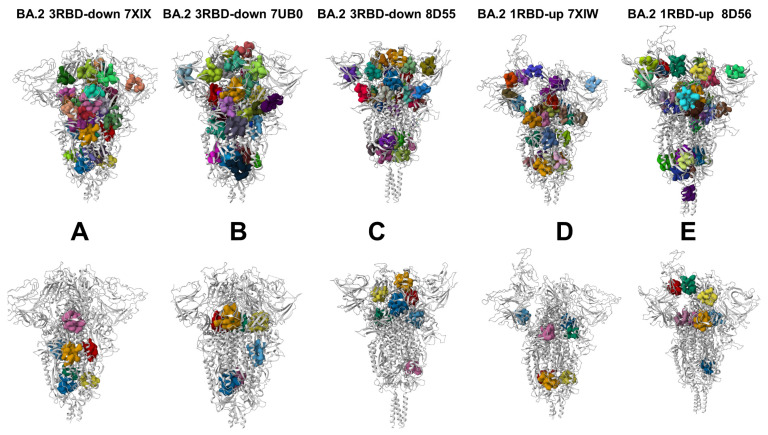
Structural maps of all identified pockets versus the top ranked cryptic sites for the ensemble of different S-BA.2 structures. Structural map of the cryptic pockets for the closed S-BA.2 trimer, pdb id 7XIX (**A**), closed BA.2 trimer, pdb id 7UB0 (**B**), closed S-BA.2 trimer, pdb id 8D55 (**C**), open BA.2 trimer, pdb id 7XIW (**D**), and open BA.2 trimer, pdb id 8D56 (**E**).

We performed a detailed quantitative analysis of the top-ranked pockets in the S-BA.1 and S-BA.2 ensembles to connect computational predictions guided by the allosteric role of the binding residues with the existing experimental data and functional knowledge of the spike mechanisms. To understand the predictive ability of the approach, evaluate potential cryptic pockets, and decipher functional relevance of these binding sites, we started by comparing structural maps of the identified binding pockets in the S protein structures against the top-ranked cryptic pockets which emerged from the conformational ensembles of the S-BA.1 and S-BA.2 structures (Figure 8 and Figure 9). First, the structural projections of all the binding pockets showed a dense network of overlapping sites. By using our approach, we characterized potential cryptic sites emerging in the ensembles that may have functional significance and could represent targetable structural elements for antibody and vaccine design purposes. Based on this premise, we first examined the top-ranked cryptic sites detected in the ensembles of different S-BA.1 structures (Figure 8). As may be expected, the predicted cryptic sites consistently emerged in the BA.1 structural ensembles (Figure 8A–E). For the S-BA.1 conformations, the top-ranked NTD cryptic pocket was predicted, along with several significant cryptic sites formed at the inter-protomer interfaces. It could be seen that the top-ranked pockets in the S-BA.1 conformations favor the S1 and S1–S2 regions, with only a fraction of the binding sites in the S2 regions (Figure 8A–E, Tables S1–S5). Given the observed structural rigidity of the closed trimers of S-BA.1, the emergence of cryptic sites in the S2 subunit indicates the underappreciated plasticity of the S2 regions.

The allosteric-guided ranking of the predicted cryptic pockets showed interesting differences between the BA.1 and BA.2 ensembles. We noticed that the NTD regions in the S-BA.2 ensembles did not harbor the most probable cryptic pockets, while these pockets were found in the RBD, S1-S2, and S2 regions (Figure 9A–E, Tables S6–S10). A more dynamic character of the open S-BA.2 trimers produced various cryptic pockets that are often specific to the conformational ensemble of a particular BA.2 trimer’s structure. To rationalize the possible functional role of the cryptic pockets as potential allosteric sites, we assumed that the network-guided pocket ranking would yield cryptic sites formed by allosterically important residues and therefore suggest plausible allosteric sites. We present a detailed analysis of the conformations of the S-BA.1 closed trimer and the top cryptic pockets (Supplementary Materials, Figure S5, Tables S1–S5). Strikingly, the top predicted NTD binding pocket (Supplementary Materials, Figure S5, pocket 1) is aligned with the experimentally discovered binding site formed by hydrophobic residues interacting with the ligand in the cryo-EM structure [55,56]. Experimental studies showed that the binding of polysorbate 80 (PS80) and the heme metabolites biliverdin and bilirubin to this NTD site can alter the epitope and modulate the antibody response [54,55,56]. Importantly, one effective NTD-targeting antibody, P008_056, competes with the small molecule biliverdin for allosterically binding to the NTD by inducing conformational changes in the loop 175–185 [55], while another effective NTD antibody, PVI.V6-14 [56], binds directly to the hydrophobic NTD pocket identified in our analysis. Our analysis captured this experimentally validated pocket and ranked it as the most probable pocket for ligand binding in the closed and open forms of the S-BA.1 trimer (Supplementary Materials, Figure S5). The latest studies highlight the conformational plasticity of the NTD regions where mutations and/or deletions not only change the architecture but also alter the surface properties, leading to a remodeling of the binding pockets, major antigenic changes in the NTD supersite, and a loss of antibody binding [57,136].

Another highly probable pocket is formed at the inter-protomer interface by the residues T274, T302, S316, and F318 of one protomer and the residues D737, C738, T739, S750, L753 L754, Y756, C760, T761, and K764 of the neighboring protomer (Supplementary Materials, Figure S5, pocket 2). Importantly, this pocket is anchored by the key hinge position F318 on one protomer and the N764 Omicron mutational site and is stabilized through the interprotomer bridges N317-D737, R319-D737, and R319-T739. Our pocket analysis highlighted as one of the five top pockets a cryptic site at the inter-protomer interface formed by the residues V551, T588, P589, C590, S591, F592, G593, Q613, and G614 of one protomer and the residues D737, M740, Y837, L841, K854, F855, K856, G857, and T859 of the neighboring protomer (Supplementary Materials, Figure S5, pocket 3). This cryptic site is stable in the conformational ensemble and is formed by critically important inter-protomer residues. This pocket is located immediately next to the ordered FPPR motif (residues 823–858) that engages in allosteric cross-talk with RBD regions [137]. Moreover, the residues K854, F855, and K856 are critical inter-protomer sites involved in inter-protomer contacts (such as N856K-D568 and N856K-T572), and changes in these positions can modulate the shifts between open and closed sites. For example, when the quadruple mutant (A570L/T572I/F855Y/N856I) introduces modifications in these positions, it can shift the equilibrium away from the closed-down state [138]. The recently developed broad-spectrum fusion inhibitors and candidate vaccines target the conserved elements in the S2 subunit, including the fusion peptide, stem helix, and HR1 and HR2 regions [95]. Our analysis identified a cryptic binding pocket in the S2 subunit formed by the residues S1037, R1039, V1040, G1046, Y1047, R1107, N1108, I909, and G910 of one protomer and the residues W886, A890, Y904, and G908 of the adjacent protomer (Supplementary Materials, Figure S5, pocket 6). This pocket is formed deeply in the S2 subunit by residues of the C-terminal β-hairpin (β_49_–β_50_) (residues 1045–1076) located downstream of the CH region. Interestingly, in several closed S-BA.1 structures (pdb id: 7TL1 and 7WK2) (Figure 8B,C) and one open S-BA.1 conformation (pdb id: 7WK3) (Figure 8D), we also identified as one of the top pockets the free fatty acid (FA) binding site [50,51,52] that bridges two RBDs of the adjacent protomers (the residues 368, 369, 372, 374, 377, 379, 384, 387, 436, 437, 440, 503, and 506 of one RBD protomer and the residues 493,405, 501, 502, 503, 504, and 505 of another RBD protomer) (Figure 8C). In agreement with the results of structural studies [50,51,52], we found that the RBD allosteric pocket may partly collapse in open BA.1 conformations. These observations indicate that the distribution of cryptic pockets may be sensitive to the conformational landscapes of the S trimers. Indeed, even for structurally similar and stable, closed BA.1 trimers, the conserved allosteric pocket between two RBDs may partly vary among different structures (Figure 8).

The pocket analysis of the S-BA.2 conformations revealed a different picture and showed evidence of a variant-specific distribution of putative allosteric sites (Supplementary Materials, Figure S6). First, the NTD binding pocket was no longer ranked as the top allosteric site as the conformational plasticity of the NTD regions and supersite was increased in the S-BA.2 conformations, causing an appreciable remodeling of the NTD binding pocket (Supplementary Materials, Figure S7). Indeed, a comparison of the NTD binding sites in different BA.1 and BA.2 conformations revealed NTD rearrangements manifesting in the fragmentation and partial occlusion of the predicted pocket in the closed and open BA.2 ensembles (Supplementary Materials, Figure S7). Our findings are consistent with recent studies of the BA.1 and BA.2 variants which showed that the neutralizing activity of anti-NTD monoclonal antibodies was markedly impaired due to the BA,2 mutation of G142D and del 143–145 in the NTD [43]. The NTD of BA1 is more remodeled compared to BA.2, presenting three substitutions, A67V, T95I, and G142D, five deleted residues (Δ69–70 and Δ143–145), and three inserted residues. As a result, the NTD-binding antibodies isolated from BA.2 do not neutralize BA.1 [139]. According to our observations, these mutational differences in the NTD between BA.1 and BA.2 can affect the conformational landscape of the NTD regions, leading to more adaptable and stable NTD binding pocket in which the respective contributing residues have significant allosteric potential. This NTD binding pocket is remodeled and is less stable in the S-BA.2 conformations (Figure 9). The sensitivity of the formation of cryptic pockets to conformational dynamics is far more pronounced for the S-BA.2 structures (Figure 9). While the pocket analysis did not detect the RBD LA pocket in some of the BA.2 conformations (Figure 9A,B), we found that this experimentally known cryptic site emerged as the most probable allosteric site for the ensemble of the closed BA.2 trimer (pdb id 8D55, Figure 9C) and was also found in the open BA.2 conformation (pdb id 8D56, Figure 9E). The top-ranked LA pocket in the BA.2 states is formed by the RBD residues F338, I358, A363, Y365, L368, Y369, F377, L387, F392, V395, C432, I434, L513, and F515 on each protomer (Supplementary Materials, Figure S6, pockets 1–3 for protomers A, C and B). A map of the LA pocket RBD residues displaying high ligand-binding propensities and mediating potential demonstrated that these sites tend to occupy other well-defined pockets on the RBD’s surface in the S-BA.2 conformations that form “pocket-based” pathways connecting the allosteric site with the RBD‘s binding interface patches (Supplementary Materials, Figure S8). The greater conformational sensitivity of the predicted cryptic pockets in the S-BA.2 structures is consistent with the dynamics analysis, which showed that the BA.2 forms are more flexible. In addition, these important findings corroborate with another computational study [140] showing that the RBD–RBD interface is less tightly packed in the closed and open BA.2 trimers. Our results confirm the notion that efficiency of allosteric LA binding to the RBD site can be determined by the Omicron variants [50,51,52,140]. In particular, we found that the LA allosteric pocket can be transiently formed in some open BA.2 conformations (Figure 9E) but is not detected in other BA.2 open states (Figure 9D). Our findings that some BA.2 closed conformations (such as the locked, closed trimer from pdb id: 8D55, Figure 9C) could prominently feature the LA binding pocket as the top-ranked site can be better understood in the context of recent functional studies [52] showing that a compact, locked 3 RBD-down S trimer is stabilized by LA occupying a bipartite binding site composed of two adjacent RBDs in the trimer. According to this study, LA binding can induce the compaction of the three RBDs, sharing characteristic features with the locked S trimer. Indeed, our predictions of the cryptic pockets in the ensemble of the S-BA.2 trimer’s conformation (Figure 9C) yielded the LA pocket as the most dominant.

In common with the S-BA.1 trimers, one of the highly ranked pockets in the closed BA.2 trimer is formed at the inter-protomer interface by the residues F541, L546, D568, A570, T572, T588, and P589 of one protomer and the residues D737, M740, Y837, L841, K854, F855, K856, G857, and T859 of the neighboring protomer (Supplementary Materials, Figure S6). A close-up comparison of this inter-protomer site between the closed S-BA.1, closed S-BA.2, and open S-BA.2 conformations showed a well-defined and conserved binding pocket in the closed states (Supplementary Materials, Figure S9). This pocket has a significant functional importance as it is formed at the inter-protomer juncture anchored by known the allosteric hotspot positions A570, T572, K854, F855, and K856 in which mutations can alter the conformational equilibrium.

The distributions for the closed S-BA.2 trimers showed a marked preference toward S2 subunit pockets, with the CH region residues in particular having the largest propensities. One of the most probable pockets is formed by the residues S1037, R1039, V1040, G1046, Y1047, R1107, N1108, I909, and G910 of one protomer and the residues W886, A890, Y904, and G908 of the adjacent protomer (Supplementary Materials, Figure S6). This pocket was also ranked highly in the S-BA.1 conformations (Supplementary Materials, Figure S5, pocket 6). Another functionally relevant cryptic pocket formed by the S2 residues of all three protomers (residues 1024, 1027, 1039, and 1042 on each protomer) presents a target for Umifenovir (Arbidol), a broad-spectrum anti-influenza drug that binds to the hydrophobic cavity of the trimer and inhibits post-fusion conformational transition related to membrane fusion [141,142]. It was proposed that Arbidol interacts with residues in the central CH-HR1 helix, the residues K776, E780, K947, E1017, R1019, S1021, N1023, L1024, and T1027 of the S2 trimerization domain [141,142], and can interfere with the trimerization of the S protein, which is critical for virus entry. We also found another highly ranked pocket composed of residues on adjacent protomers and overlapping with the highly conserved, conformational hinge epitope, which spans residues 980–1006 of the S protein, at the apex of the S2 domain [143]. The discovery of these pockets that overlap or are immediately adjacent to the S2 hinge epitope are important in light of the findings that antibody evasion may occur through changes in the dynamics of the neighboring regions that impact the accessibility of the hinge epitope. The hinge epitope and neighboring cryptic pockets are protected by tightly packed RBDs in the down state, but access to these pockets may be modulated through global dynamic changes, and long-range couplings between the S1 and S2 subunits can affect the formation and stability of the cryptic binding sites.

We also mapped the critical network clusters obtained in the dynamic network analysis with the determined distribution of cryptic binding sites (Supplementary Materials, Figure S10). By identifying local residue clusters that correspond to the major peaks of the network distributions (Figure 5), we inferred the positions of regions harboring potential allosteric hotspot points that mediate long-range interactions. In this model, we assumed that these clusters would overlap with the putative allosteric binding sites. According to the network analysis, the key inter-protomer interfaces are associated with the bridging communities linking neighboring protomers (B-855 C-589 C-592; A-568 A-574 C-854; A-740 A-857 B-592; B-775 B-864 C-665; A-592 C-737 C-855). At the same time, the pocket analysis predicted the formation of a cryptic binding site by the residues V551, T588, P589, C590, S591, F592, G593, Q613, and G614 of one protomer and the residues D737, M740, Y837, L841, K854, F855, K856, G857, and T859 of the neighboring protomer (Supplementary Materials, Figure S5, pocket 3). This pocket becomes well-structured and stable in the conformational ensemble and is overlapped with one of the key network clusters which is important for mediating allosteric communications in the S trimer. Hence, the predicted inter-protomer pocket may represent a functionally important allosteric site where targeting may directly interfere with the hinge movements of the S1 subunit with respect to S2 and impair conformational transitions to the open form (Supplementary Materials, Figure S10). In the RBD regions, a group of hydrophobic, high-centrality centers from the RBD core form a well-defined conserved allosteric binding pocket corresponding to the experimentally determined allosteric site where the essential free fatty acid LA binds. The structural mapping of the high-centrality residue clusters connecting the S2 pockets with the inter-protomer allosteric pocket and the RBD’s allosteric binding sites demonstrated a densely packed network, especially in the closed form (Supplementary Materials, Figure S10). Moreover, the structural maps indicate the importance of the networking bridges N764K-Q314, S982-T547K, N856K-D568, N856K-T572, N969K-Q755, N969K-Y756, S383-D985, and F855-G614 (in black spheres, Supplementary Materials, Figure S10), which provide “stepping stones” for long-range communication and connectivity between distant functional regions and allosteric sites.

## 4. Discussion

Our study leveraged the growing wealth of closed and open structures for the S-BA.1 and S-BA.2 trimers to explore the conformational landscapes of these S proteins and examine the evolution and characteristics of cryptic binding pockets in these ensembles. Despite considerable structural similarities between the closed 3RBD-down structures, the conformational landscapes revealed some specific characteristics that were translated into a rich distribution of the identified cryptic pockets. Using an allosteric-based network ranking of the binding pockets, we recovered all the experimentally known allosteric sites and discovered significant variant-specific differences in the distribution of cryptic binding sites in the BA.1 and BA.2 trimers. While both closed and open S-BA.1 trimers featured as highly ranked in the NTD supersite region [63], the conformational dynamics of the S-BA.2 trimers could partly mask the NTD’s cryptic region and present previously underappreciated cryptic binding pockets in the inter-protomer interface and hinge regions of the S2 subunit. The in-depth analysis of the conformation-specific evolution of the NTD pockets highlighted the notion that mutational changes in the NTD between BA.1 and BA.2 are translated into the conformational adaptability and variant-specific remodeling of the NTD site. Mutational changes in the BA.1 variant can induce a druggable NTD pocket that is preserved and ranked as the most probable in both closed and open BA.1 conformations. This result is particularly interesting as it suggests that despite the conformational plasticity afforded in the NTD, this allosteric pocket is preserved and accessible for ligand binding in BA.1 conformations. At the same time, our findings suggested that the distinct mutational signature in the NTDs of the BA.2 trimers may result in considerable remodeling and fragmentation of the NTD binding pocket. Interestingly, our studies highlighted that the NTD’s allosteric binding sites harbor variable regions and can undergo considerable remodeling between Omicron variants [61], whereas the evolutionary conservation of the LA-binding pocket in the Omicron variants [52] can co-exist with the partial restructuring and adaptability of the RBD-RBD. Our predictions of the conformation and variant-specific preferences of the LA pocket are in line with studies on LA binding with the S protein which show scenarios of dynamic opening–closing of the pocket through gating helix motions or the opening of the pocket while interacting with LA [52]. We argue that the opening/closing of the LA pocket is linked with the conformational plasticity of the S trimers, particularly for the BA.2 variant. As a result, LA binding may occur through a conformational selection of a closed or open conformational state in which the RBD-RBD pocket is accessible, followed by an LA-induced stabilization of the locked, closed form. The observed variant-sensitive adaptability of the RBD-RBD allosteric site could also affect how structural changes in the RBD are propagated to other functional sites while preserving the general topology of the LA binding pocket in spike.

Understanding of the interplay of conformational dynamics changes induced by Omicron variants and the identification of cryptic dynamic binding pockets in the S protein are of paramount importance in COVID-19 research as exploring broad-spectrum antiviral agents to combat the emerging variants is imperative. In contrast to a significant accumulation of mutations within the RBD, the S2 fusion subunit has remained highly conserved among variants, with only several mutational sites targeted by the Omicron variants. Identifying druggable sites in the S2 subunit can present new and previously underappreciated opportunities for therapeutic intervention given the increasing number of fusion inhibitors and candidate vaccines that target the conserved elements in the S2 subunit [112,113]. The results of our study suggest that despite the general rigidity of the S2 regions in comparison with the more dynamic S1 subunit, there is still an appreciable level of conformational adaptability in the S2, resulting in a significant number of dynamic cryptic pockets in the S2. In particular, our data point to several conserved pockets in the HR1 and CH regions of the rigid S2 subunit, thereby indicating that an appreciable level of plasticity is present in the S2 regions, giving rise to the broader accumulation of dynamic cryptic pockets. The results of this study provide a molecular rationale and support to the experimental evidence that the acquisition of functionally balanced substitutions that optimize multiple fitness tradeoffs between immune evasion, host binding affinity, and sufficient conformational adaptability might be a common strategy in evolution of the virus and serve as a primary driving force behind the emergence of new Omicron subvariants.

## 5. Conclusions

In the current study, we explored conformational landscapes and characterized the universe of cryptic binding pockets in multiple open and closed functional spike states of the Omicron variants BA.1 and BA.2. Conformational dynamics and network analyses confirmed that the S-BA.1 and S-BA.2 trimers may display different levels of conformational plasticity and dynamic changes, allowing for the modulation of the opening and closing cryptic binding pockets in the S1 and S2 subunits. By using a combination of atomistic simulations, a dynamics network analysis, and an allostery-guided network screening of cryptic pockets in the conformational ensembles of the BA.1 and BA.2 variants’ spike conformations, we demonstrated that the proposed approach would recover the experimentally known allosteric sites in the NTD, RBD, and targetable S2 regions as highly ranked top binding pockets. The results of this study indicate that while both closed and open S-BA.1 trimers featured as highly ranked in the NTD supersite region, the conformational dynamics of the S-BA.2 trimers could mask the NTD’s cryptic region and present previously underappreciated cryptic binding pockets in the inter-protomer interface and hinge regions of the S2 subunit. We found that mutational differences in the NTD between BA.1 and BA.2 can affect the conformational landscape of the NTD regions, leading to more adaptable and stable NTD binding pockets in the BA.1 structures, while the NTD binding pockets are remodeled and less stable in the S-BA.2 conformations. The central result of this analysis is the discovery of variant-specific differences in the distribution of cryptic binding sites in the BA.1 and BA.2 trimers, suggesting that small variations could lead to different preferences in the allocation of druggable sites. The predicted conformation and variant-specific preferences of the RBD allosteric pocket agree with the observed dependency of LA binding to the RBD site between the Omicron variants and the binding preference toward locked, closed S conformations. The determined cryptic binding pockets at the inter-protomer regions and in the functional regions of the S2 subunit, such as the HR1-HR2 bundle and stem helix region, are consistent with the roles of the pocket residues in modulating conformational transitions and antibody recognition. Of particular interest is the detection of highly probable pockets in the S2 regions known to be allosterically linked with the RBD’s movements. The results of this study are particularly significant for understanding the universe of cryptic bindings sites and the detection of conformation-dependent, variant-specific preferences for conserved, druggable pockets. Targeted ligand screening in the predicted druggable sites can allow for the design of variant-specific modulators of the S protein’s activity and the ligand-induced stabilization of specific conformational states, thus providing chemical tools for systematically probing the S protein’s functions and binding. The results of this study provide a better understanding of the interplay between the conformational plasticity and evolution of novel druggable cryptic pockets in S trimers, suggesting the diversity of targetable regions and the variant-specific preferences of allosteric pockets. This may enable the engineering of efficient and selective modulators that could rationally target a complex functional landscape of virus transmissibility.

## Figures and Tables

**Table 1:** Mutational landscape of the Omicron BA.1 and BA.2 mutations.

Omicron Variant	Mutational Landscape
BA.1	A67, T95I, G339D, S371L, S373P, S375F, K417N, N440K, G446S, S477N, T478K, E484A, Q493R, G496S, Q498R, N501Y, Y505H, T547K, D614G, H655Y, N679K, P681H, N764K, D796Y, N856K, Q954H, N969K, and L981F
BA.2	T19I, G142D, V213G, G339D, S371F, S373P, S375F, T376A, D405N, R408S, K417N, N440K, S477N, T478K, E484A, Q493R, Q498R, N501Y, Y505H, D614G, H655Y, N679K, P681H, N764K, D796Y, Q954H, and N969K

**Table 2:** Structures of the SARS-CoV-2 S Omicron protein structures examined in this study.

Variant	PDB Code	Description	RBD Orientation	Resolution	Ref.
BA.1	7TF8	S Omicron Trimer, closed	3 RBD-down	3.9	[27]
BA.1	7WK2	S Omicron Trimer, closed	3 RBD-down	3.1	[24]
BA.1	7TNW	S Omicron Trimer, closed	3 RBD-down	3.1	[37]
BA.1	7TL1	S Omicron Trimer, closed	3 RBD-down	3.5	[27]
BA.1	7TL9	S Omicron Trimer, open	1 RBD-up	3.5	[27]
BA.1	7TEI	S Omicron Trimer, open	1 RBD-up	3.4	[27]
BA.1	7WK3	S Omicron Trimer, open	1 RBD-up	3.4	[24]
BA.1	7TO4	S Omicron Trimer, open	1 RBD-up	3.4	[37]
BA.1	7WVN	S Omicron Trimer, open	1 RBD-up	4.0	[24]
BA.1	7WVO	S Omicron Trimer, open	1 RBD-up	4.0	[24]
BA.1	7TGE	S Omicron Trimer, open	2 RBD-up	3.7	[27]
BA.2	7XIX	S Omicron Trimer, closed	3 RBD-down	3.25	[38]
BA.2	7UB0	S Omicron Trimer, closed	3 RBD-down	3.31	[31]
BA.2	7UB5	S Omicron Trimer, closed	3 RBD-down	3.35	[31]
BA.2	7UB6	S Omicron Trimer, closed	3 RBD-down	3.52	[31]
BA.2	8D55	S Omicron Trimer, closed	3 RBD-down	2.8	[37]
BA.2	7XIW	S Omicron Trimer, open	1 RBD-up	3.62	[38]
BA.2	8D56	S Omicron Trimer, open	1 RBD-up	3.0	[37]

**Table 3:** Molecular simulations of RBD-ACE2 complexes.

PDB	System		CG-BD	# Simulations	All-Atom MD
7WK2	S-BA.1	3-RBD down	500,000 steps	100	500 ns
7WK3	S-BA.1	1-RBD up	500,000 steps	100	500 ns
7XIX	S-BA.2	3-RBD down	500,000 steps	100	500 ns
7XIW	S-BA.2	1-RBD up	500,000 steps	100	500 ns

# stands for number of simulations.

## Data Availability

The data are fully contained within the article. The crystal structures were obtained and downloaded from the Protein Data Bank (http://www.rcsb.org, accessed 17 April 2023). All simulations were performed using the NAMD 2.13 package, which was obtained from website https://www.ks.uiuc.edu/Development/Download/ (accessed 10 May 2023). All simulations were performed using the all-atom additive CHARMM36 protein force field, which can be obtained from http://mackerell.umaryland.edu/charmm_ff.shtml (accessed 10 May 2023). The residue interaction network files were obtained for all structures using the Residue Interaction Network Generator (RING) program, RING v2.0.1, which is freely available at http://old.protein.bio.unipd.it/ring/ (accessed 10 May 2023). The computations of the network parameters were carried out using Cytoscape 3.8.2, which is available at https://cytoscape.org/download.html (accessed 20 May 2023). The rendering of the protein structures was completed using the interactive visualization program UCSF ChimeraX package (https://www.rbvi.ucsf.edu/chimerax/, accessed 22 April 2023) and Pymol (https://pymol.org/2/, accessed 22 April 2023).

## Supplementary Materials

The following supporting information can be downloaded at: https://www.mdpi.com/article/10.3390/v15102009/s1. Table S1: The list and pocket scores for the top pockets in the S-BA.1 3RBD-down (pdb id 7TF8).; Table S2: The list and pocket scores for the top pockets in the S-BA.1 3RBD-down (pdb id 7TL1); Table S3: The list and pocket scores for the top pockets in the S-BA.1 3RBD-down (pdb id 7WK2); Table S4: The list and pocket scores for the top pockets in the S-BA.1 1RBD-up (pdb id 7WK3); Table S5: The list and pocket scores for the top pockets in the S-BA.1 2RBD-up (pdb id 7TGE); Table S6: The list and pocket scores for the top pockets in the S-BA.2 3RBD-down (pdb id 7XIX); Table S7: The list and pocket scores for the top pockets in the S-BA.2 3RBD-down (pdb id 7UB0); Table S8: The list and pocket scores for top pockets in the S-BA.2 3RBD-down (pdb id 8D55); Table S9: The list and pocket scores for the top pockets in the S-BA.2 1RBD-up (pdb id 7XIW); Table S10: The list and pocket scores for the top pockets in the S-BA.2 1RBD-up (pdb id 8D56). Figure S1: Principal component analysis (PCA) of the conformational ensembles for the S-BA.1 and S-BA.2 trimers; Figure S2: The slow-mode dynamics profiles represent the displacements along slow-mode eigenvectors and correspond to the contribution of the five slowest modes; Figure S3: Structural maps of the conformational dynamics profiles corresponding to the cumulative contribution of five slowest low-frequency modes for the Omicron S-BA.1 and S-BA.2 trimers; Figure S4: Structural maps of the conformational dynamics profiles corresponding to the slowest low-frequency mode for the Omicron S-BA.1 and S-BA.2 trimers; Figure S5: Structural map and residue-based close-ups of the top ranked cryptic sites for the ensemble of the S-BA.1 closed trimers; Figure S6: Structural map and residue-based close-ups of the top-ranked cryptic sites for the ensemble of the S-BA.2 closed trimers. Figure S7: A comparison of the predicted NTD binding pockets in the closed BA.1 trimer structures, BA.2 closed trimer structures, and BA.2 open trimer conformations; Figure S8: A comparison of the predicted RBD binding pocket for the BA.1 closed conformation and BA.2 closed conformations; Figure S9: A comparison of the predicted inter-protomer cryptic pocket in the BA.1 closed trimers, BA.2 closed trimers, and BA.2 open trimer conformations. Figure S10: Structural mapping of the network-based communication paths and inter-protomer network bridges between allosteric binding sites in the S Omicron trimer structures.
