# Supplementary material for: Exploring Conformational Landscapes and Cryptic Binding Pockets in Distinct Functional States of the SARS-CoV-2 Omicron BA.1 and BA.2 Trimers: Mutation-Induced Modulation of Protein Dynamics and Network-Guided Prediction of Variant-Specific Allosteric Binding Sites
Source: Viruses. 2023 Sep 27;15(10):2009. doi: 10.3390/v15102009 (PMC10610873; doi:10.3390/v15102009)
Supplement: Supplementary file 1 [file viruses-15-02009-s001.zip › SUPPLEMENTARY_MATERIALS/SUPPLEMENTARY_MATERIALS_VIRUSES_REVISION.docx]

Supplementary Materials

Exploring Conformational Landscapes and Cryptic Binding Pockets in Distinct Functional States of the SARS-CoV-2 Omicron BA.1 and BA.2 Trimers : Mutation-Induced Modulation of Protein Dynamics and Network-Guided Prediction of Variant-Specific Allosteric Binding Sites

Gennady Verkhivker,^1,2^* Mohammed Alshahrani,^1^ Grace Gupta^1^

^1^ Keck Center for Science and Engineering, Graduate Program in Computational and Data Sciences, Schmid

College of Science and Technology, Chapman University, Orange, CA 92866, United States of

America

^2^ Department of Biomedical and Pharmaceutical Sciences, Chapman University School of Pharmacy, Irvine, CA 92618, United States of America

***** Correspondence: verkhivk@chapman.edu; Tel.: +1-714-516-4586 (G.V)


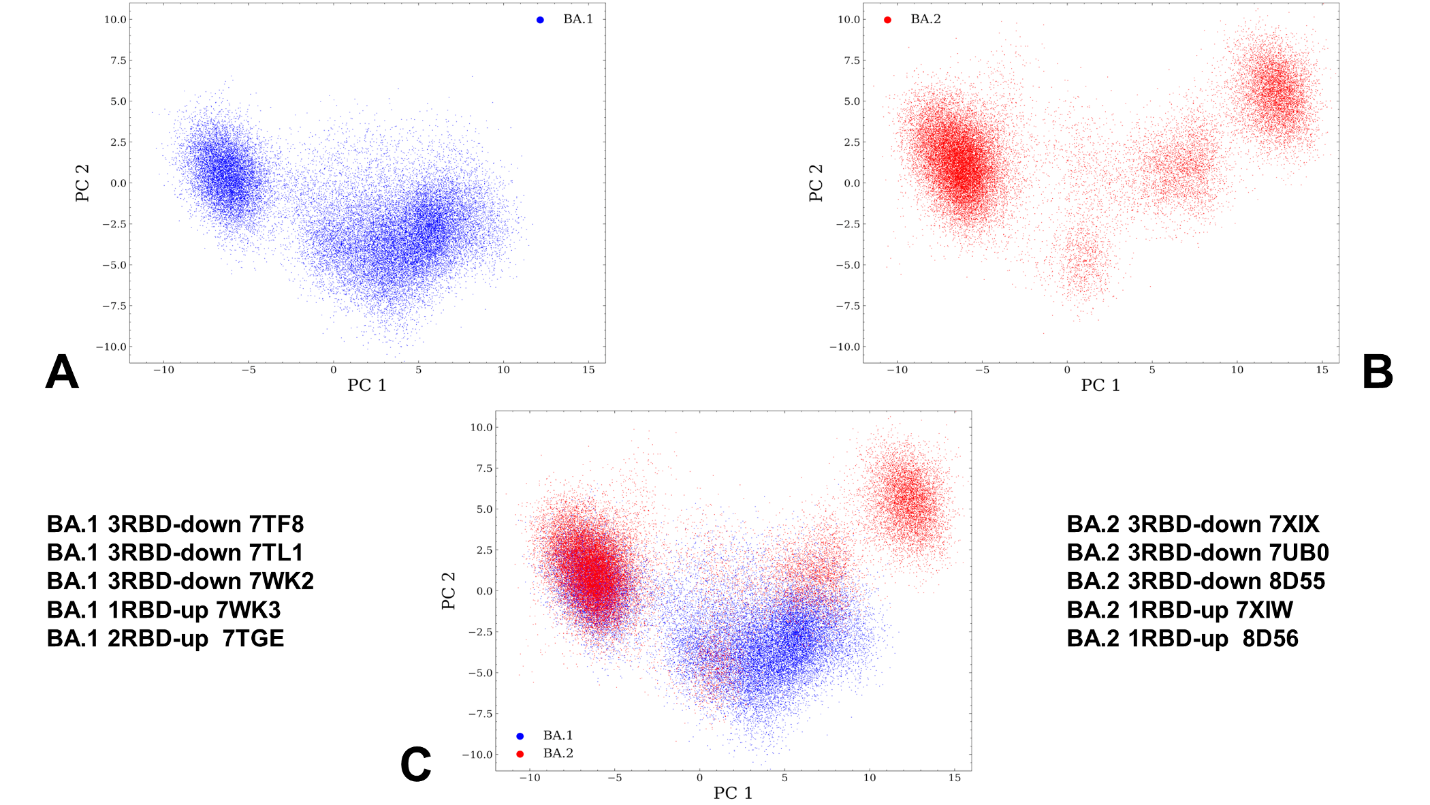


**Figure S1.** Principal component analysis (PCA) of the conformational ensembles for the S-BA.1 and S-BA.2 trimers. The trajectories of the molecular simulations were analyzed using principal component analysis (PCA), which separates out the motions of the S protein for BA.1 and BA.2 trimers into principal modes ranked according to their relative contributions. (A) PCA of the trajectories and low-dimensional density on of the conformational space obtained from simulations of S-BA.1 trimers (pdb id 7TF8, 7TL1, 7WK2, 7WK3, 7TGE) in blue dots. (B) PCA of the trajectories and low-dimensional density of the conformational space obtained from simulations of S-BA.2 trimers (pdb id 7XIX, 7UB0, 8D55, 7XIW, 8D56) shown in red dots. (C) Overlap of low-dimensional density on of the conformational space obtained from simulations of S-BA.1 trimers (in blue dots) and S-BA.2 trimers (in red dots).


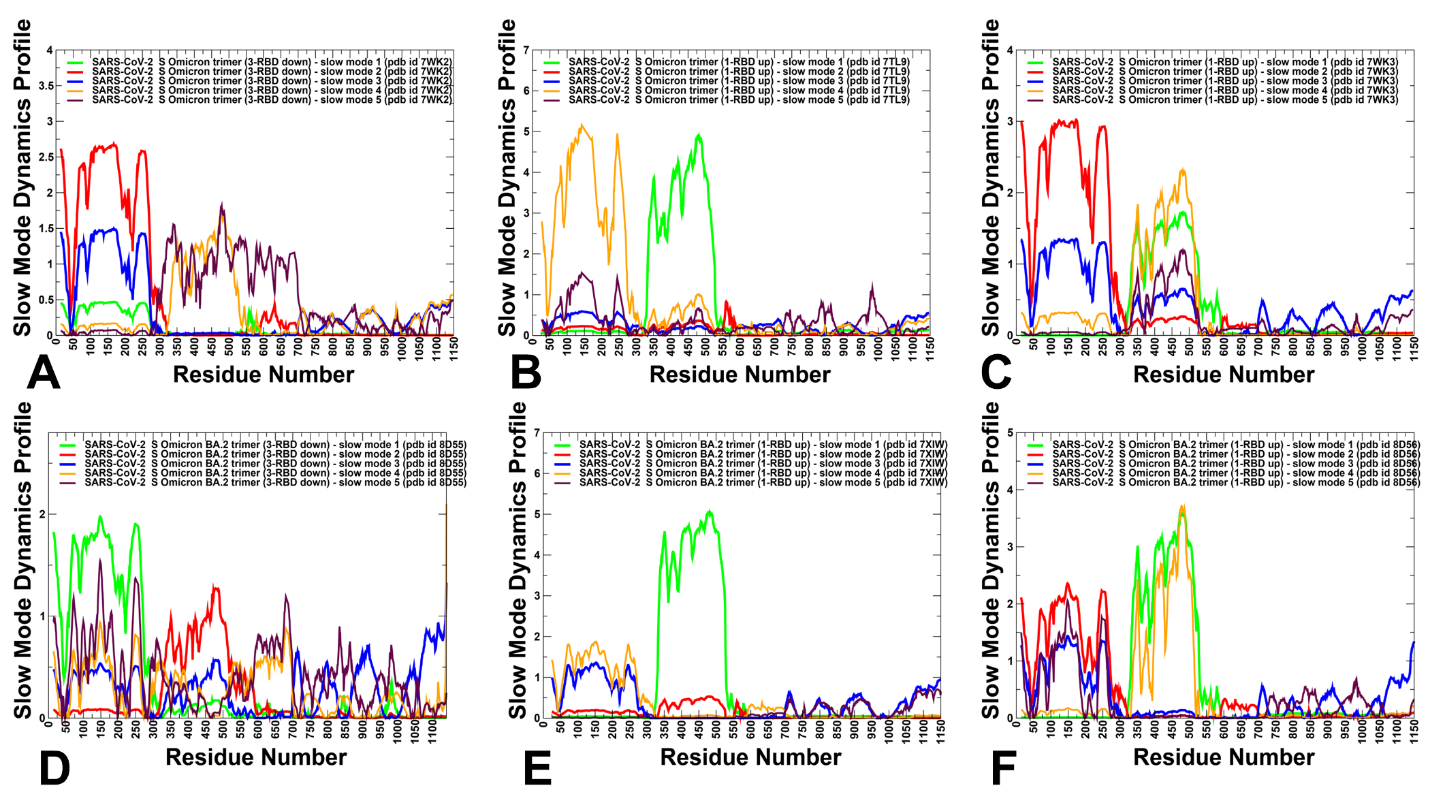


**Figure S2.** The slow mode mobility profiles of the S Omicron trimer structures. The slow mode dynamics profiles represent the displacements along slow mode eigenvectors and correspond to the contribution of the five slowest modes. The slow mode mobility profiles for the S-BA.1 trimer states : 3RBD-down closed BA.1 trimer, pdb id 7WK2 (A), 1RBD-up open BA.1 trimer, pdb id 7TL9 (B), and 1RBD-up open BA.1 trimer, pdb id 7WK3 (C). The slow mode mobility profiles for the S-BA.2 trimer states: 3RBD-down closed BA.2 trimer, pdb id 8D55 (D), 1RBD-up open BA.2 trimer, pdb id 7XIW (E), and 1RBD-up open BA.2 trimer, pdb id 8D56 (F). The slow mode profiles are shown for mode 1 (in green lines), mode 2( in red lines), mode 3 (in blue lines), mode 4 (in orange lines) and mode 5 (in maroon-colored lines).


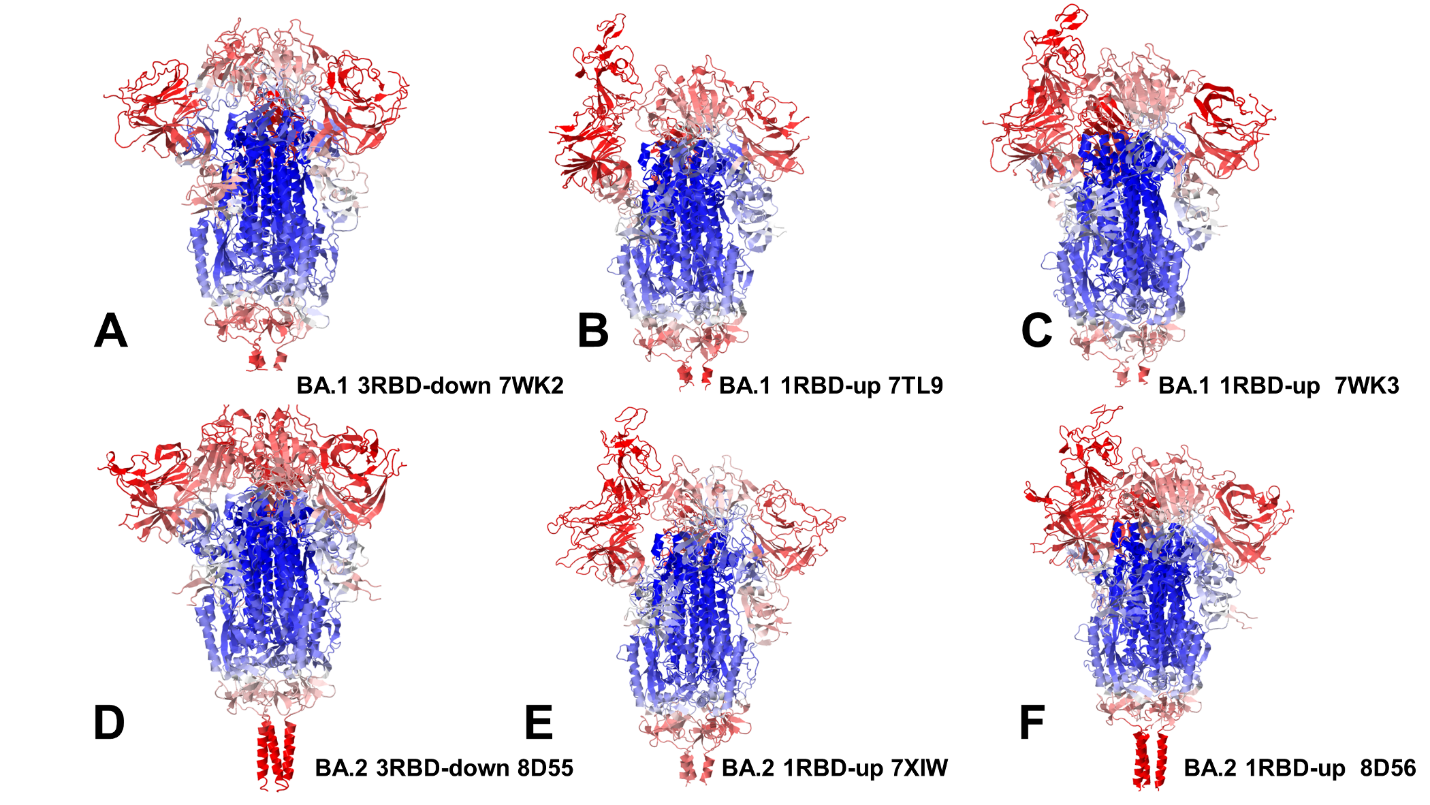


**Figure S3.** Structural maps of the conformational dynamics profiles corresponding to the cumulative contribution of five slowest low-frequency modes for Omicron S-BA.1 and S-BA.2 trimers. Conformational mobility maps are shown for the BA.1 3RBD-down trimer, pdb id 7WK2 (A), BA. 1RBD-up trimer, pdb id 7TL9 (B), BA.1 1RBD-up trimer, pdb id 7WK3 (C), BA.2 3RBD-down trimer, pdb id 8D55 (D), BA.2 1RBD-up trimer, pdb id 7XIW (E), and BA.2 1RBD-up trimer (F). The structures are shown in ribbons with the rigidity-flexibility sliding scale colored from blue (most rigid) to red (most flexible).


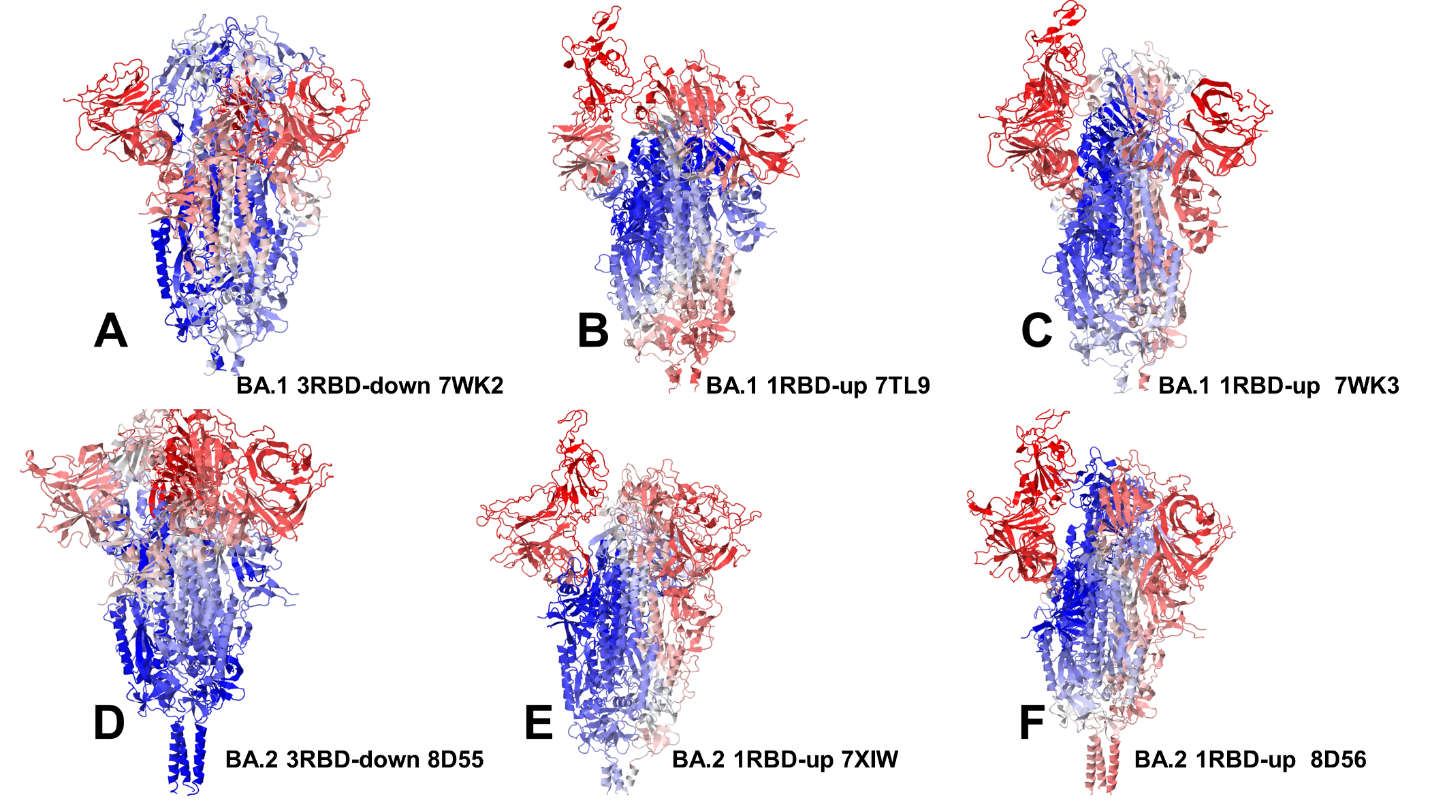


**Figure S4.** Structural maps of the conformational dynamics profiles corresponding to the slowest low-frequency mode for Omicron S-BA.1 and S-BA.2 trimers. Conformational mobility maps are shown for the BA.1 3RBD-down trimer, pdb id 7WK2 (A), BA. 1RBD-up trimer, pdb id 7TL9 (B), BA.1 1RBD-up trimer, pdb id 7WK3 (C), BA.2 3RBD-down trimer, pdb id 8D55 (D), BA.2 1RBD-up trimer, pdb id 7XIW (E), and BA.2 1RBD-up trimer (F). The structures are shown in ribbons with the rigidity-flexibility sliding scale colored from blue (most rigid) to red (most flexible).


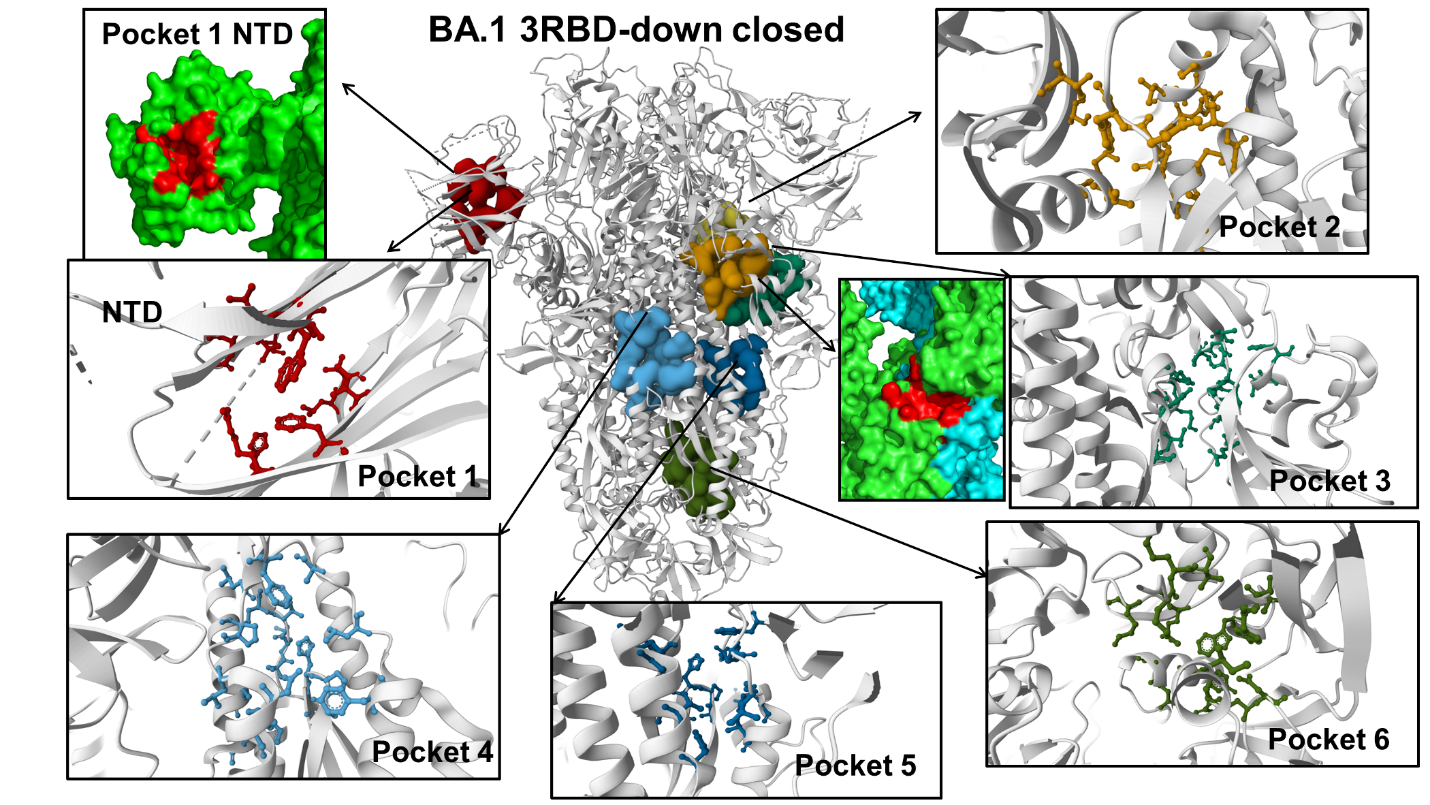


**Figure S5.** Structural map and residue-based close-ups of the **top ranked cryptic sites** for the ensemble of the S-BA.1 closed trimers. The BA.1 closed trimer (pdb id 7TF8) is shown in ribbons and the top ranked allosteric sites are in surface. Pocket 1 ( residues 101, 104, 119, 121, 126, 128, 170, 172, 190, 192, 194, 203, 205, 207, 226, 227 in each of the protomer). Pocket 1 is also highlighted in red surface with NTD in green. Pocket 2 (residues B_274, B_302, B_304, B_316, B_317, B_318, B_50, C_738, C_739, C_750, C_753, C_754, C_756, C_757, C_760, C_761, C_764). Pocket 3 (residues B_551, B_588, B_589, B_590, B_591, B_592, B_593, B_594, B_613, B_622, B_623, B_625, C_735, C_737, C_740, C_837, C_841, C_854, C_855, C_857, C_859). Pocket 3 is also shown in red surface at the inter-protomer interface of two protomers ( in green and cyan surface). Pocket 4 (residues B_1056, B_1057, B_1058, B_1059, B_730, B_731, B_732, B_733, B_778, B_782, B_823, B_828, B_831, B_833, B_861, B_863, B_865, B_867, B_870). Pocket 5 (**residues** C_1056, C_1057, C_1058, C_1059, C_729, C_730, C_731, C_774, C_778, C_782, C_823, C_828, C_833, C_863, C_865, C_867, C_870). Pocket 6 (residues B_1037, B_1039, B_1040, B_1046, B_1047, B_1107, B_1108, B_909, B_910, C_1036, C_1038, C_886, C_890, C_904, C_907, C_908)


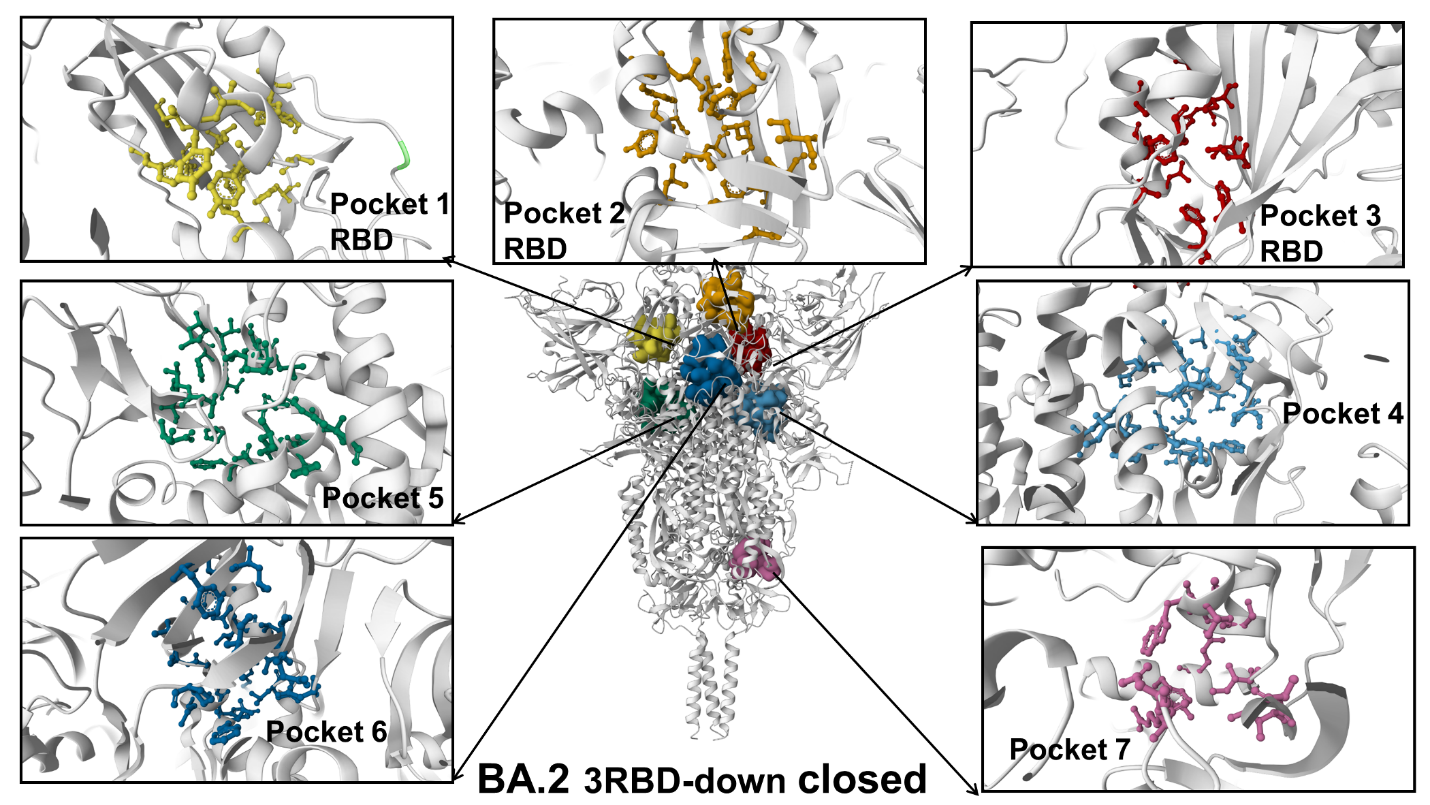


**Figure S6.** Structural map and residue-based close-ups of the top ranked cryptic sites for the ensemble of the S-BA.2 closed trimers. The BA.2 closed trimer (pdb id 8D55) is shown in ribbons and the top ranked allosteric sites are in surface. Pocket 1 ( residues A_338, A_363, A_365, A_368, A_374, A_377, A_387, A_392, A_395, A_432, A_434, A_513, A_515, protomer A). Pocket 2 (residues C_338, C_358, C_363, C_365, C_368, C_369, C_377, C_387, C_392, C_395, C_432, C_434, C_513, C_515, protomer C). Pocket 3 (residues B_338, B_342, B_358, B_363, B_365, B_368, B_369, B_377, B_387, B_392, B_395, B_432, B_434, B_513, B_515, protomer B). Pocket 4 (residues A_541, A_546, A_548, A_549, A_568, A_570, A_572, A_573, A_574, A_587, A_588, A_589, C_1000, C_740, C_741, C_742, C_744, C_745, C_845, C_852, C_855, C_856, C_966, C_975, C_976, C_977, C_978). Pocket 5 (residues B_1000, B_740, B_741, B_742, B_744, B_745, B_855, B_856, B_966, B_975, B_976, B_977, B_978, C_541, C_546, C_547, C_548, C_549, C_568, C_572, C_573, C_587, C_589). Pocket 6 ( residues A_1000, A_740, A_741, A_742, A_744, A_745, A_855, A_856, A_966, A_975, A_976, A_977, A_978, B_541, B_546, B_547, B_548, B_549, B_568, B_570, B_572, B_573, B_574, B_587, B_589). Pocket 7 (residues A_1107, A_1108, A_712, A_713, C_884, C_885, C_886, C_887, C_896, C_901, C_904).


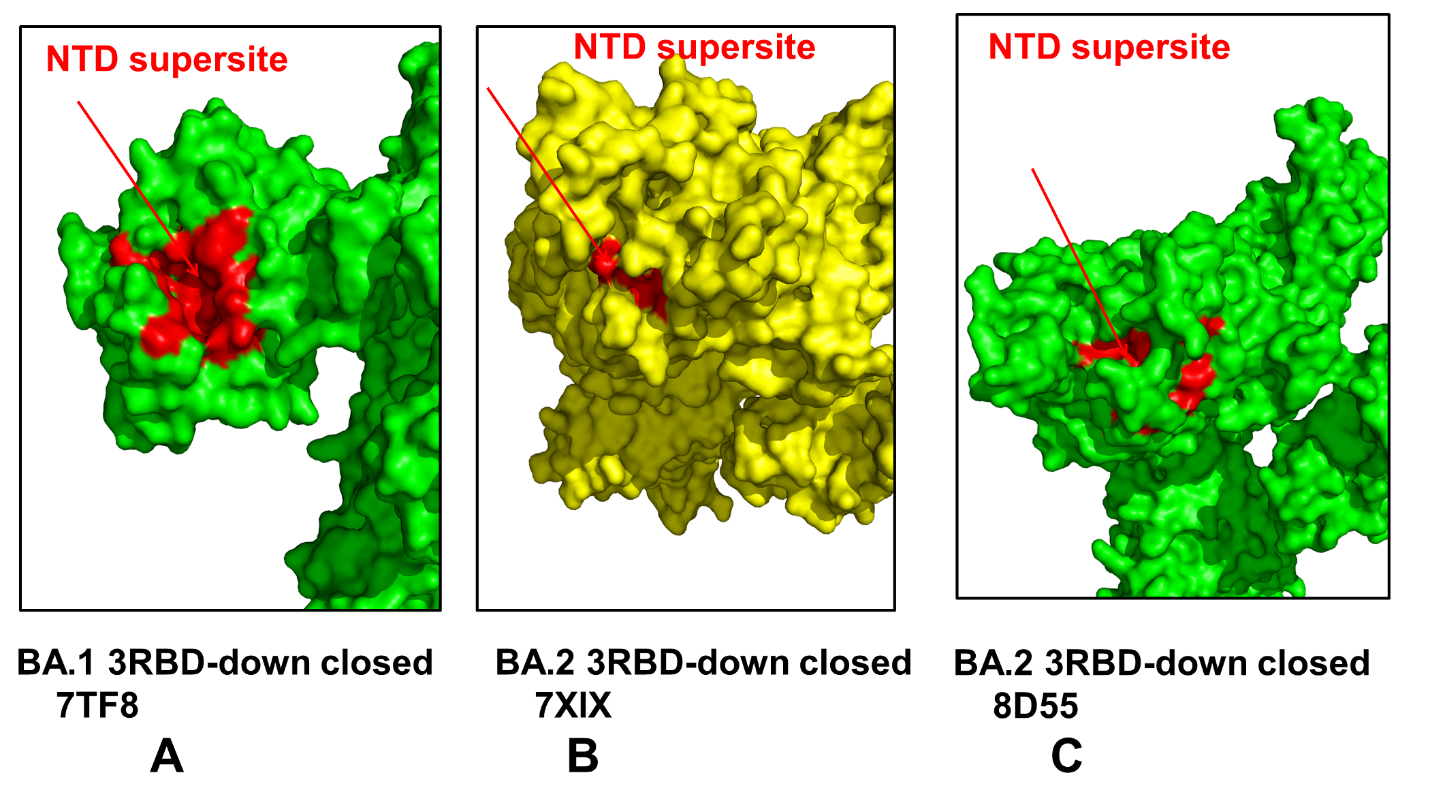


**Figure S7.** A comparison of the predicted NTD binding pockets in the closed BA.1 trimer structures (A), BA.2 closed trimer structures (B) and BA.2 open trimer conformations (C). The S protein is shown in green surface. The predicted NTD binding pocket is shown in red surface and indicated by arrow. The predicted NTD pocket residues overlap with the experimentally known NTD supersite formed by residues 14–20, residues 140–158 (the supersite b-hairpin), and residues 245–264 (the supersite loop).


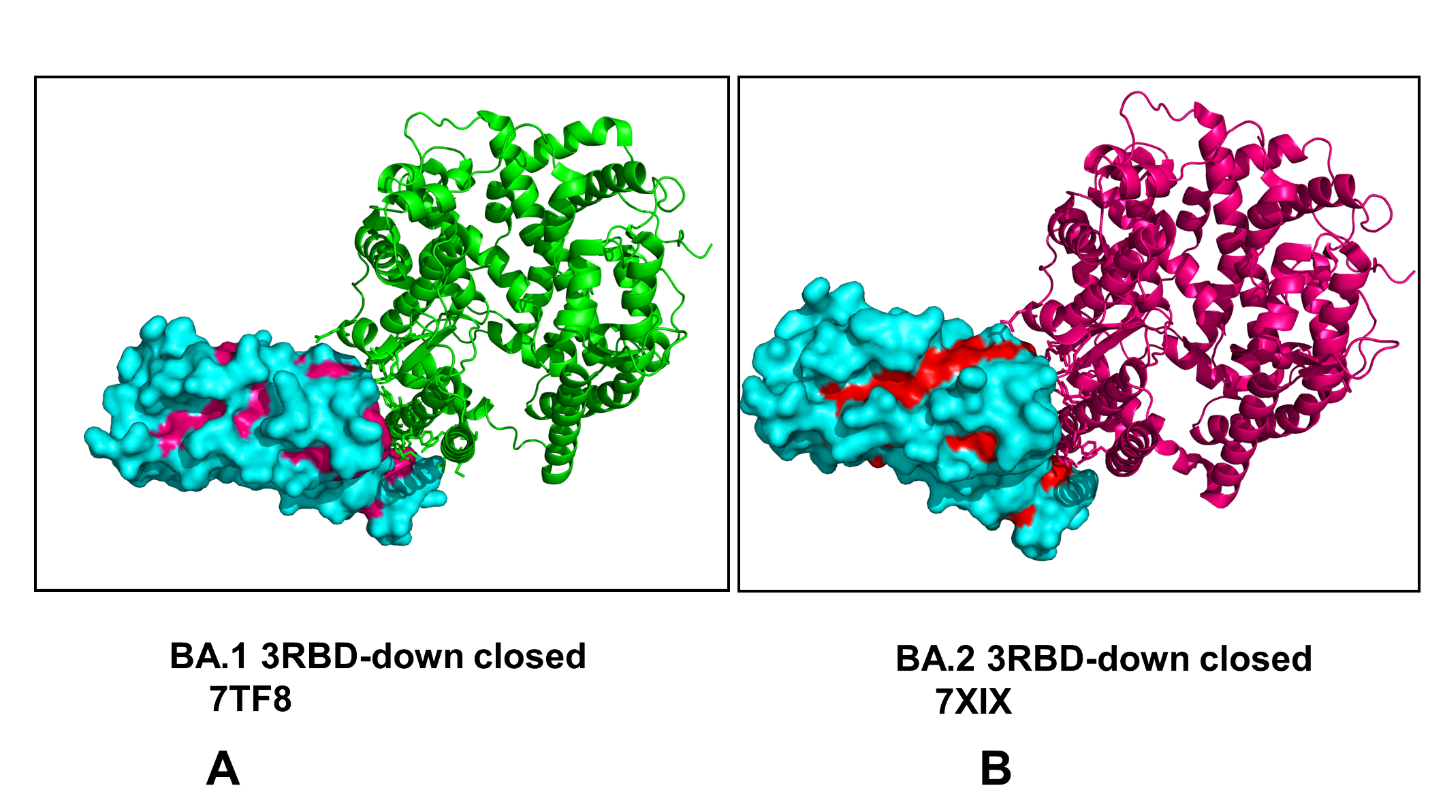


**Figure S8.** A comparison of the predicted RBD binding pocket for the BA.1 closed conformations (A) and BA.2 closed conformations (B). The RBD is shown in cyan surface, the RBD pocket regions formed are shown in pink surface (A) and red surface (B). The host receptor ACE2 is shown in green ribbons (A) and dark pink (B)


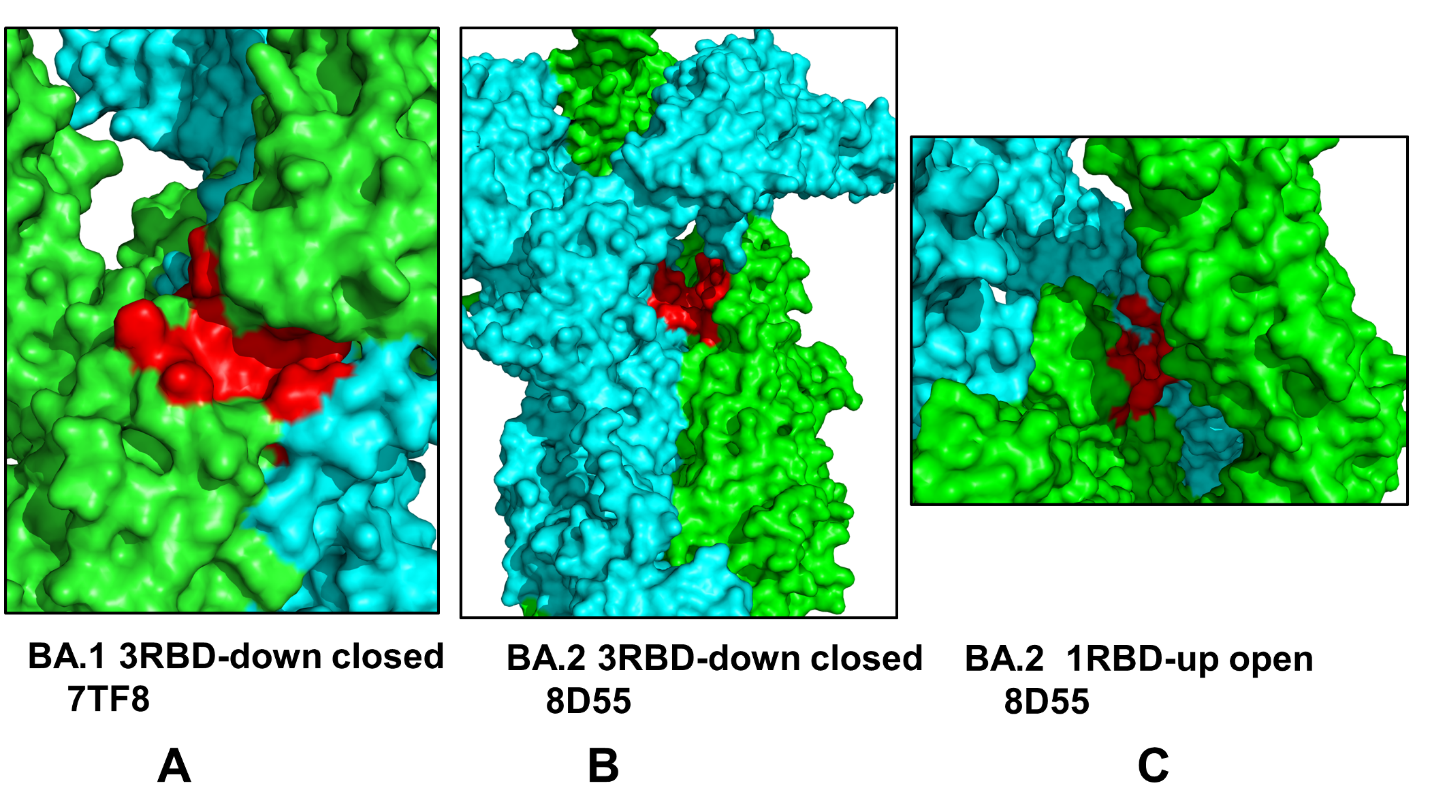


**Figure S9.** A comparison of the predicted inter-protomer cryptic pocket in the BA.1 closed trimers (A), BA.2 closed trimers (B) and BA.2 open trimer conformations (C). The S protein protomers are shown in green and cyan surface. The predicted cryptic pocket is shown in red surface. This cryptic site at the inter-protomer interface is formed by residues V551, T588, P589, C590, S591, F592, G593, Q613, G614 of one protomer and residues D737, M740, Y837, L841, K854, F855, K856, G857 and T859 of the neighboring protomer.


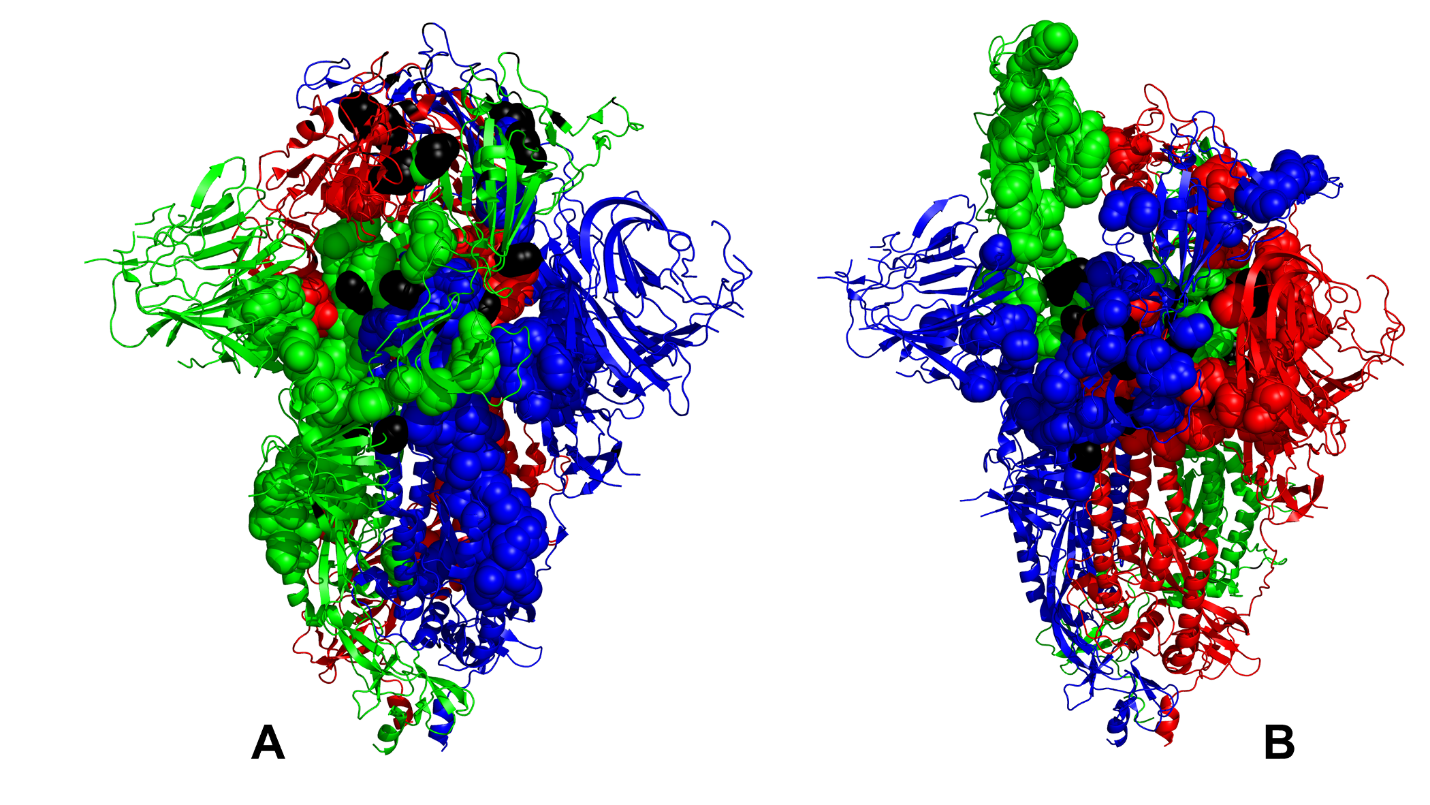


**Figure S10.** Structural mapping of the network-based communication paths and inter-protomer network bridges between allosteric binding sites (S2, S1-S2 inter-protomer hinge site and RBD allosteric site) in the 3-RBD down closed S Omicron trimer structures (A) and 1RBD-up open S Omicron trimer structures. The Omicron trimer is shown in ribbons with the protomers A,B,C in green, red and blue respectively. The inter-protomer bridges are shown in black spheres.
