# Supplementary figures and images for: Exploring Conformational Landscapes and Cryptic Binding Pockets in Distinct Functional States of the SARS-CoV-2 Omicron BA.1 and BA.2 Trimers: Mutation-Induced Modulation of Protein Dynamics and Network-Guided Prediction of Variant-Specific Allosteric Binding Sites
Source: Viruses. 2023 Sep 27;15(10):2009. doi: 10.3390/v15102009 (PMC10610873; doi:10.3390/v15102009)

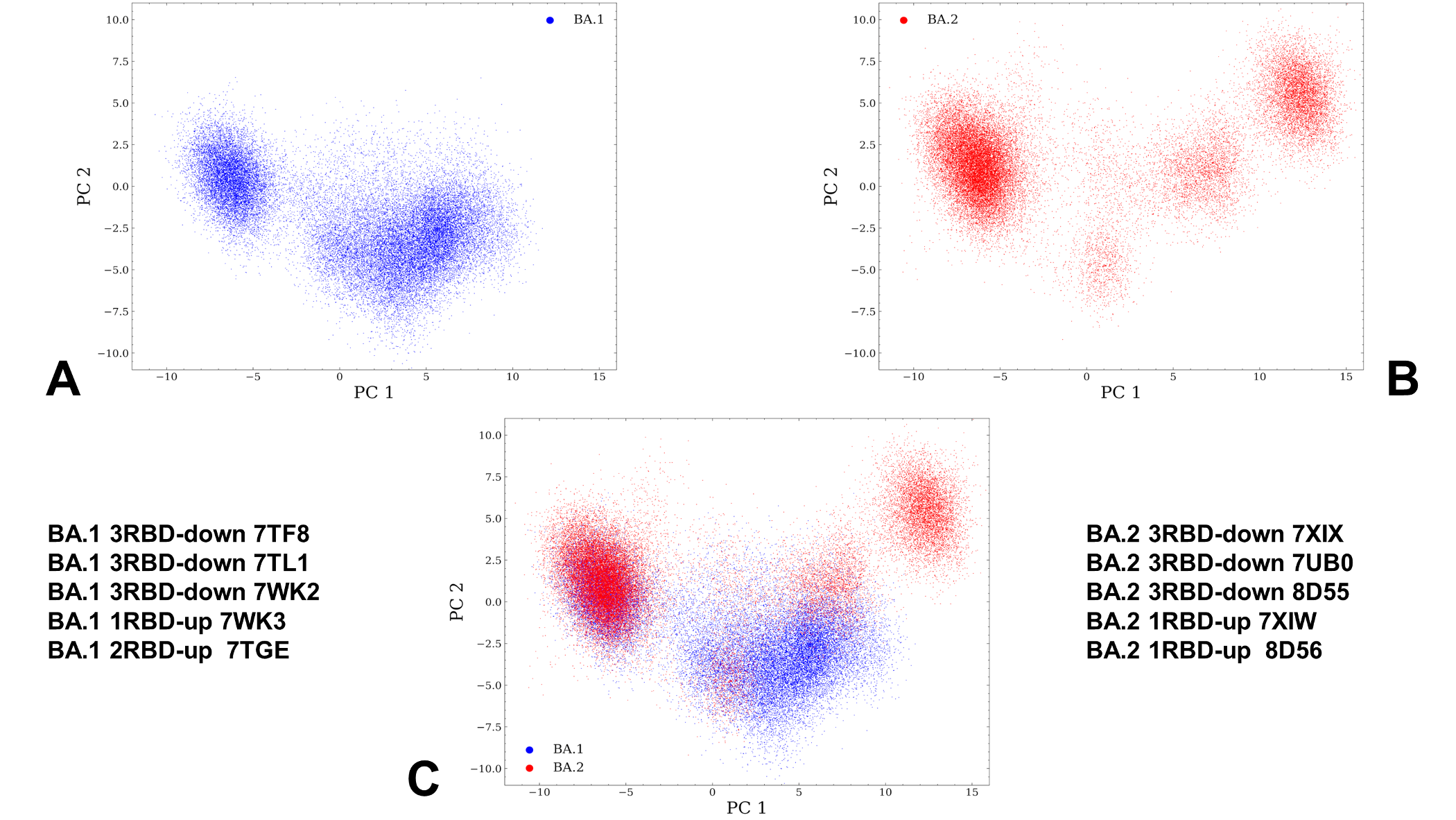

Supplement: Supplementary file 1 [file viruses-15-02009-s001.zip › SUPPLEMENTARY_MATERIALS/FigureS1.tif]

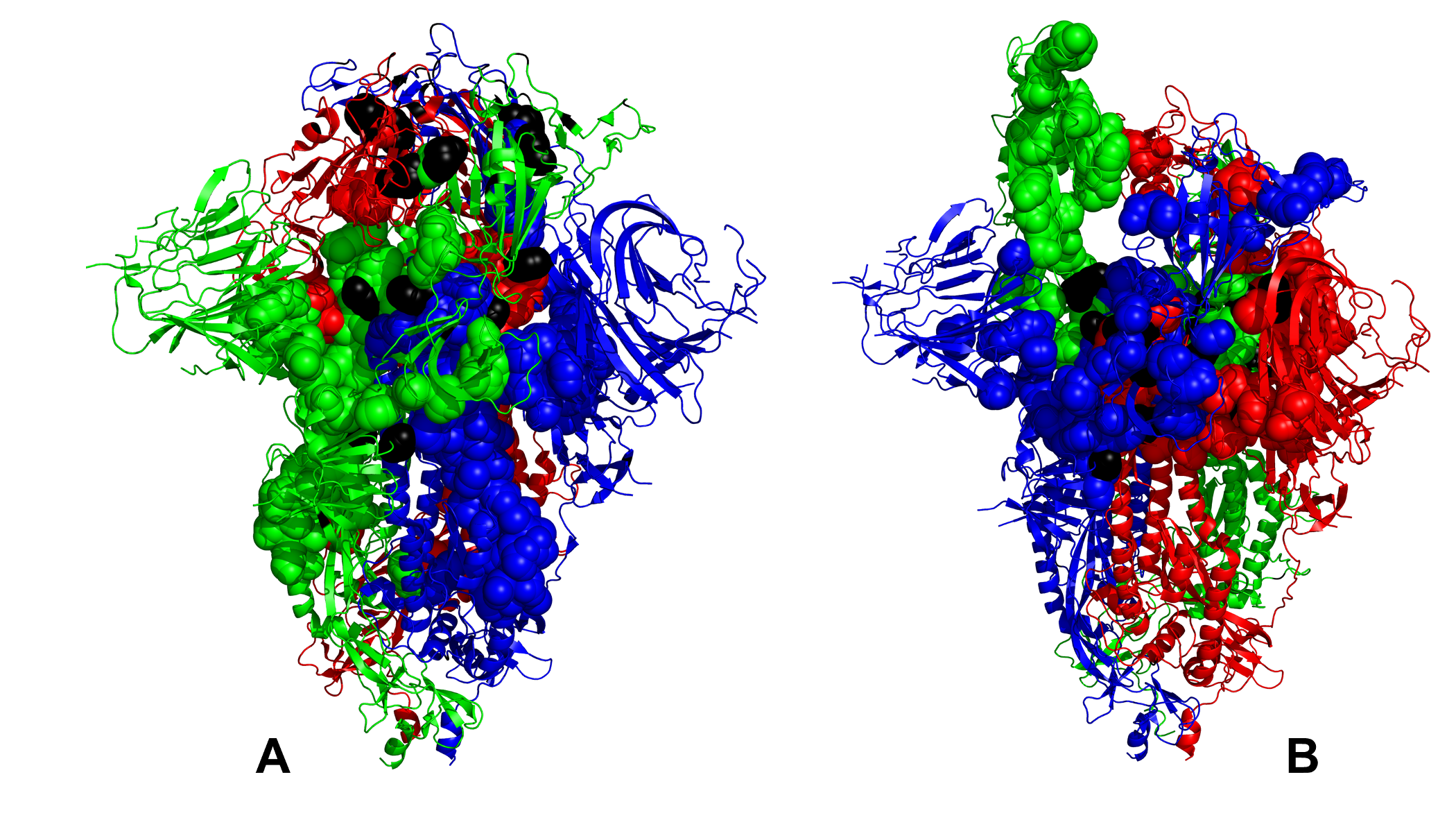

Supplement: Supplementary file 1 [file viruses-15-02009-s001.zip › SUPPLEMENTARY_MATERIALS/FigureS10.tif]

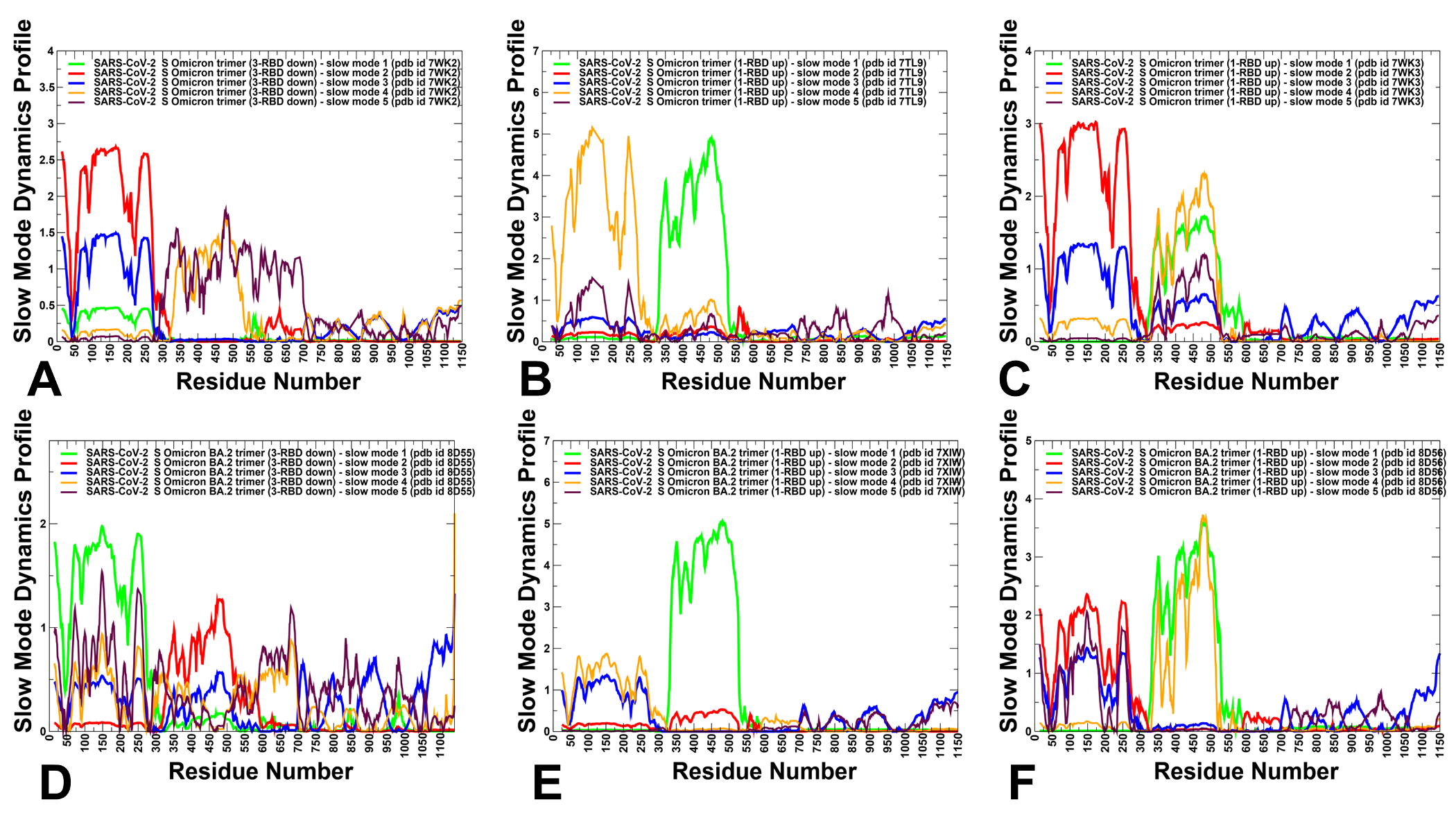

Supplement: Supplementary file 1 [file viruses-15-02009-s001.zip › SUPPLEMENTARY_MATERIALS/FigureS2.tif]

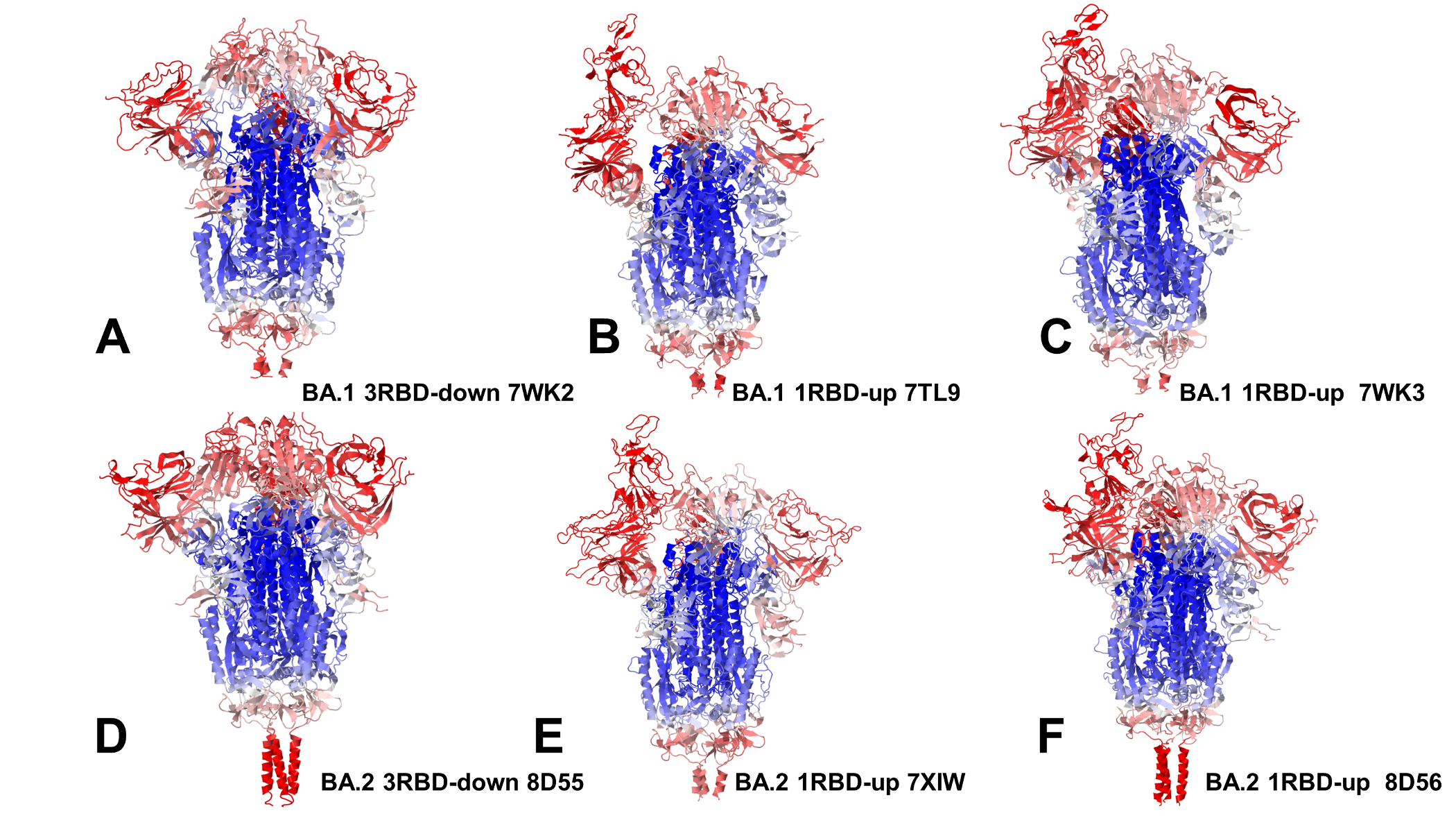

Supplement: Supplementary file 1 [file viruses-15-02009-s001.zip › SUPPLEMENTARY_MATERIALS/FigureS3.tif]

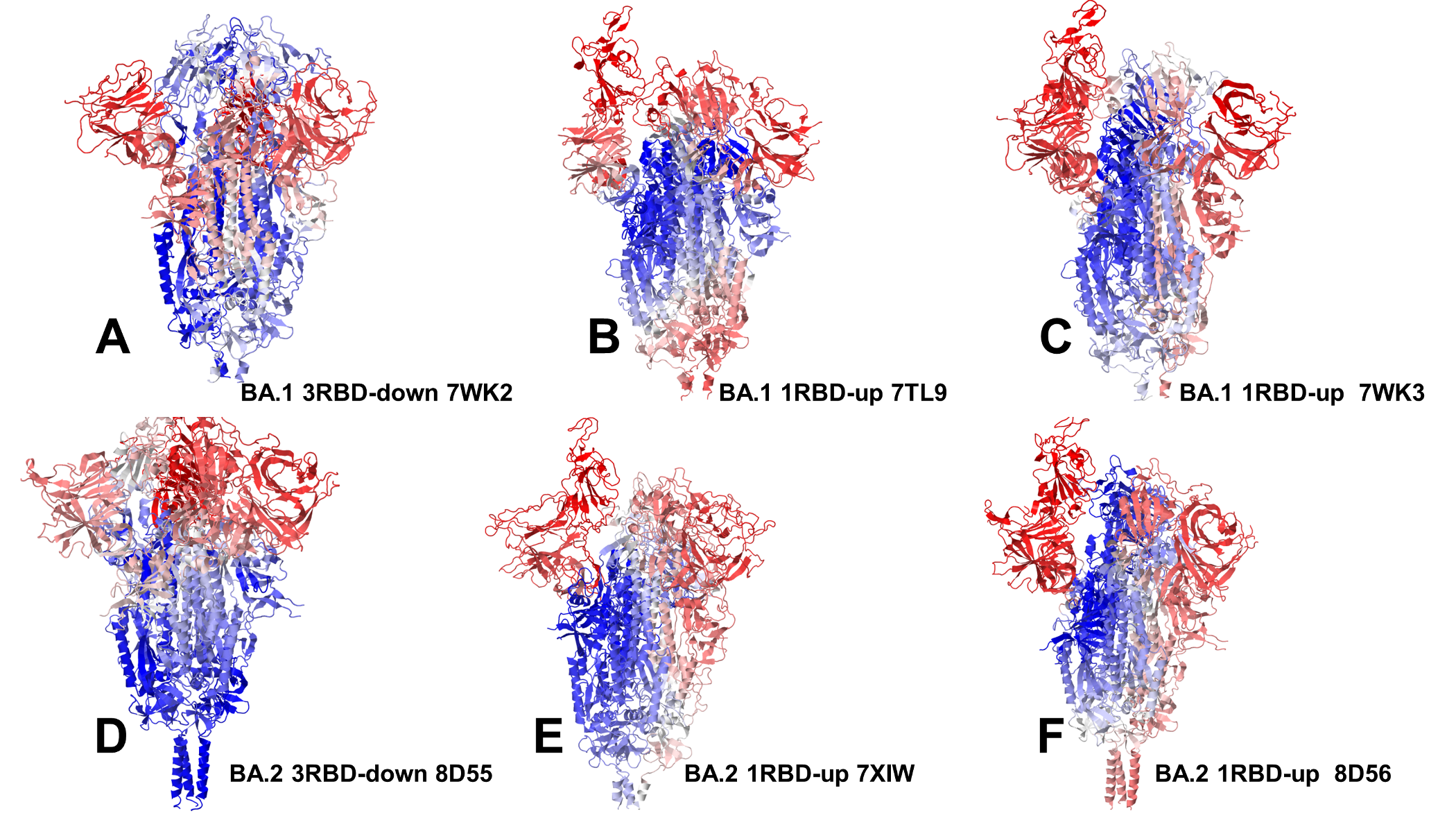

Supplement: Supplementary file 1 [file viruses-15-02009-s001.zip › SUPPLEMENTARY_MATERIALS/FigureS4.tif]

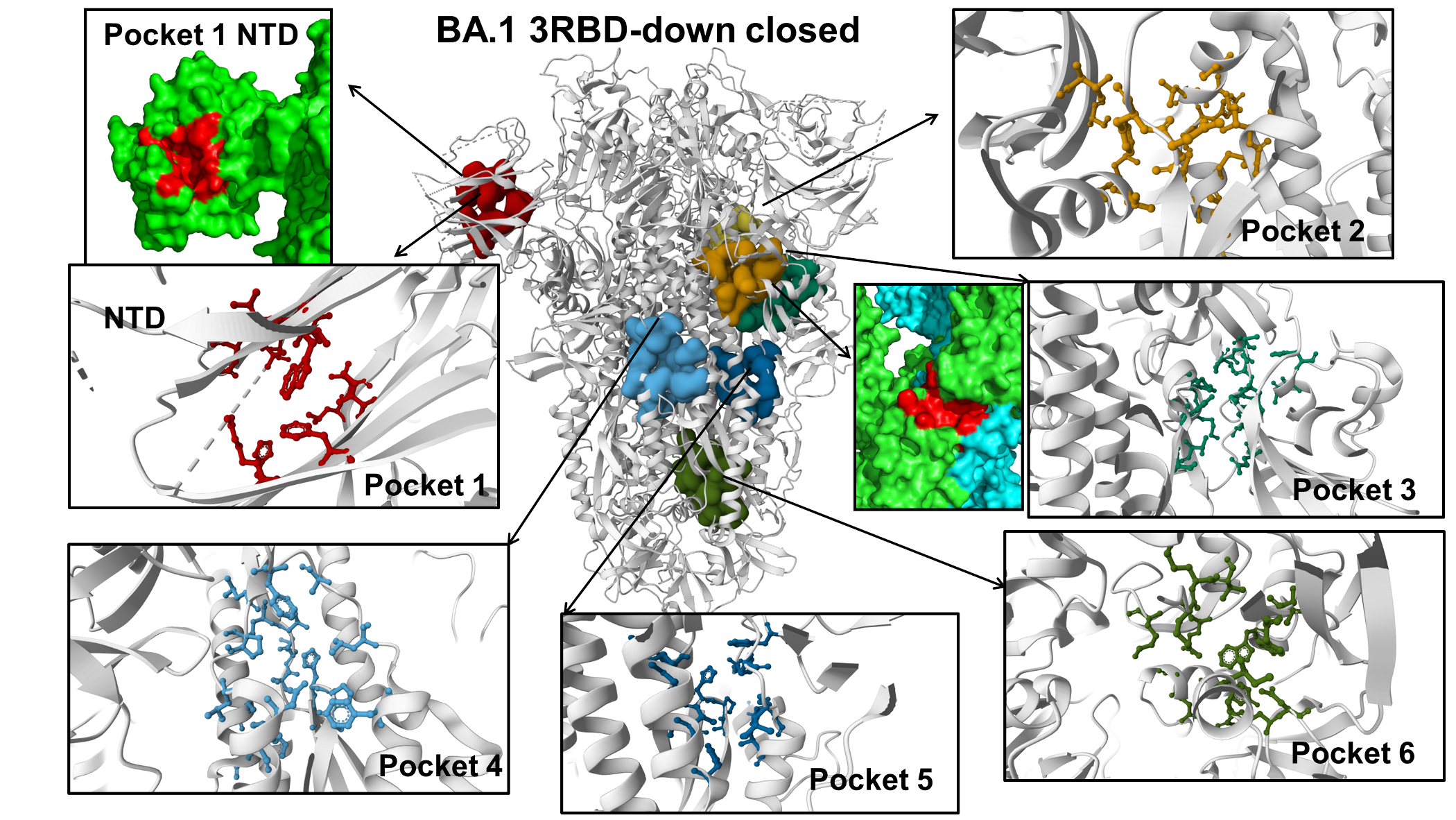

Supplement: Supplementary file 1 [file viruses-15-02009-s001.zip › SUPPLEMENTARY_MATERIALS/FigureS5.tif]

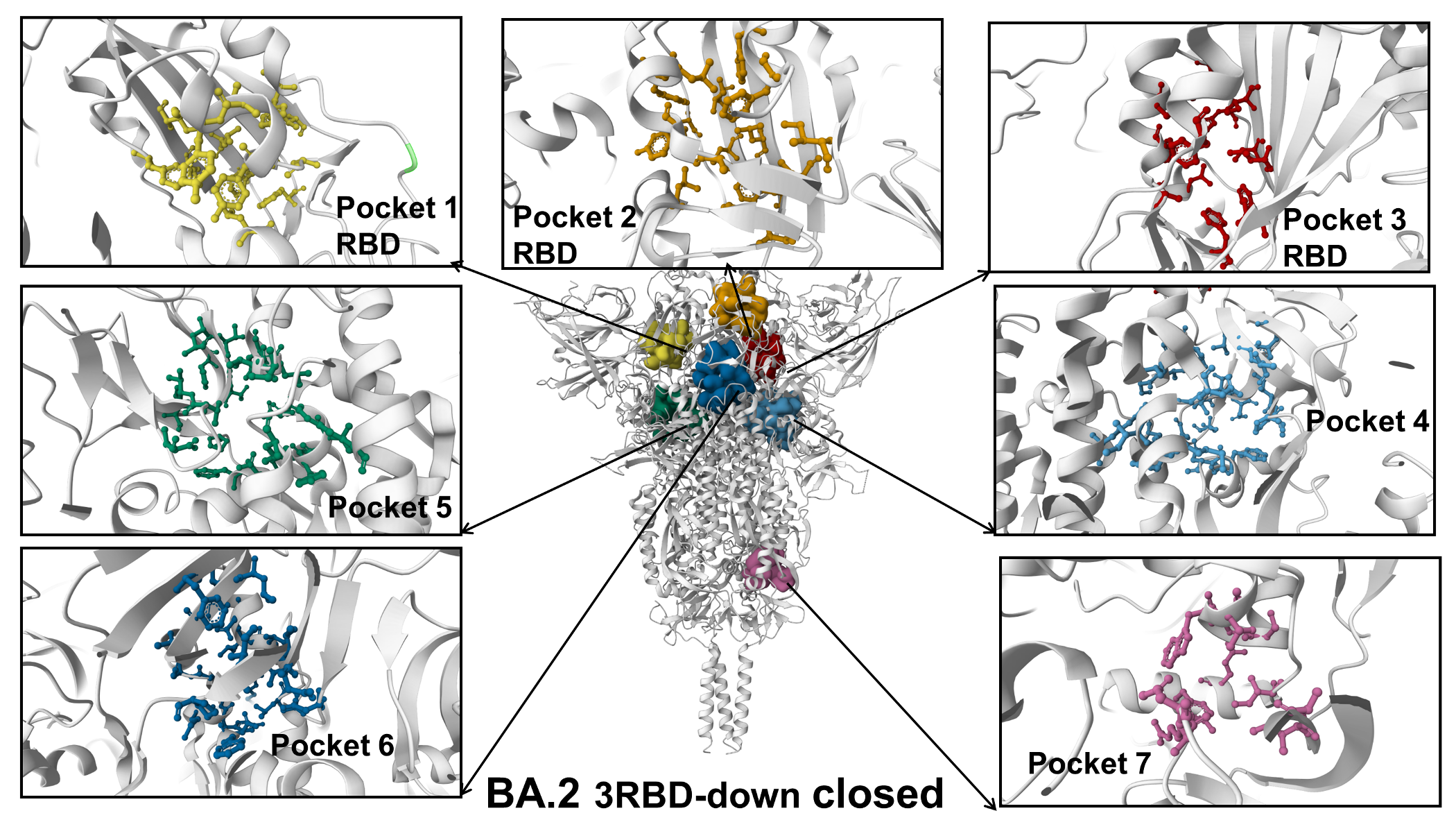

Supplement: Supplementary file 1 [file viruses-15-02009-s001.zip › SUPPLEMENTARY_MATERIALS/FigureS6.tif]

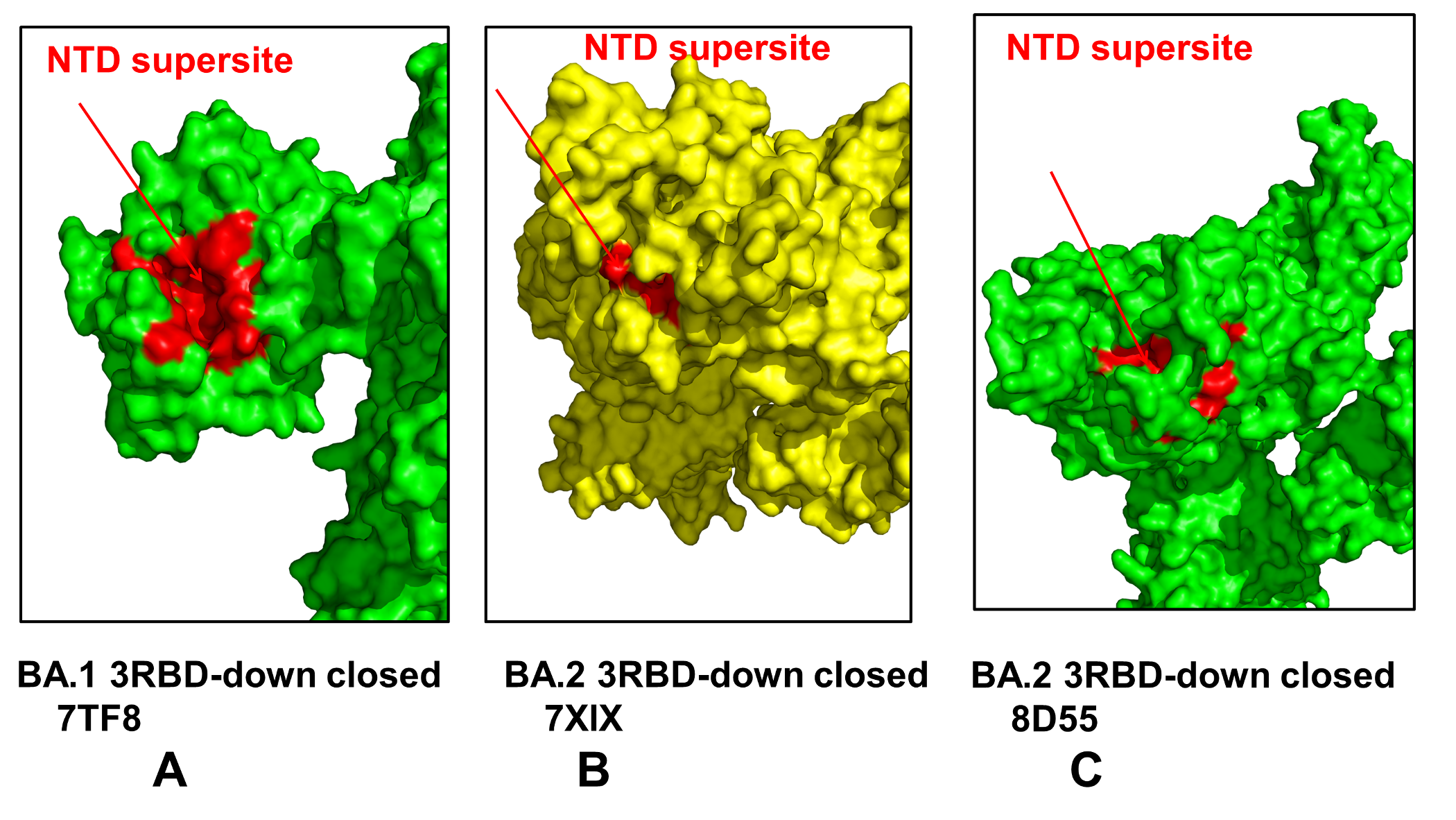

Supplement: Supplementary file 1 [file viruses-15-02009-s001.zip › SUPPLEMENTARY_MATERIALS/FigureS7.tif]

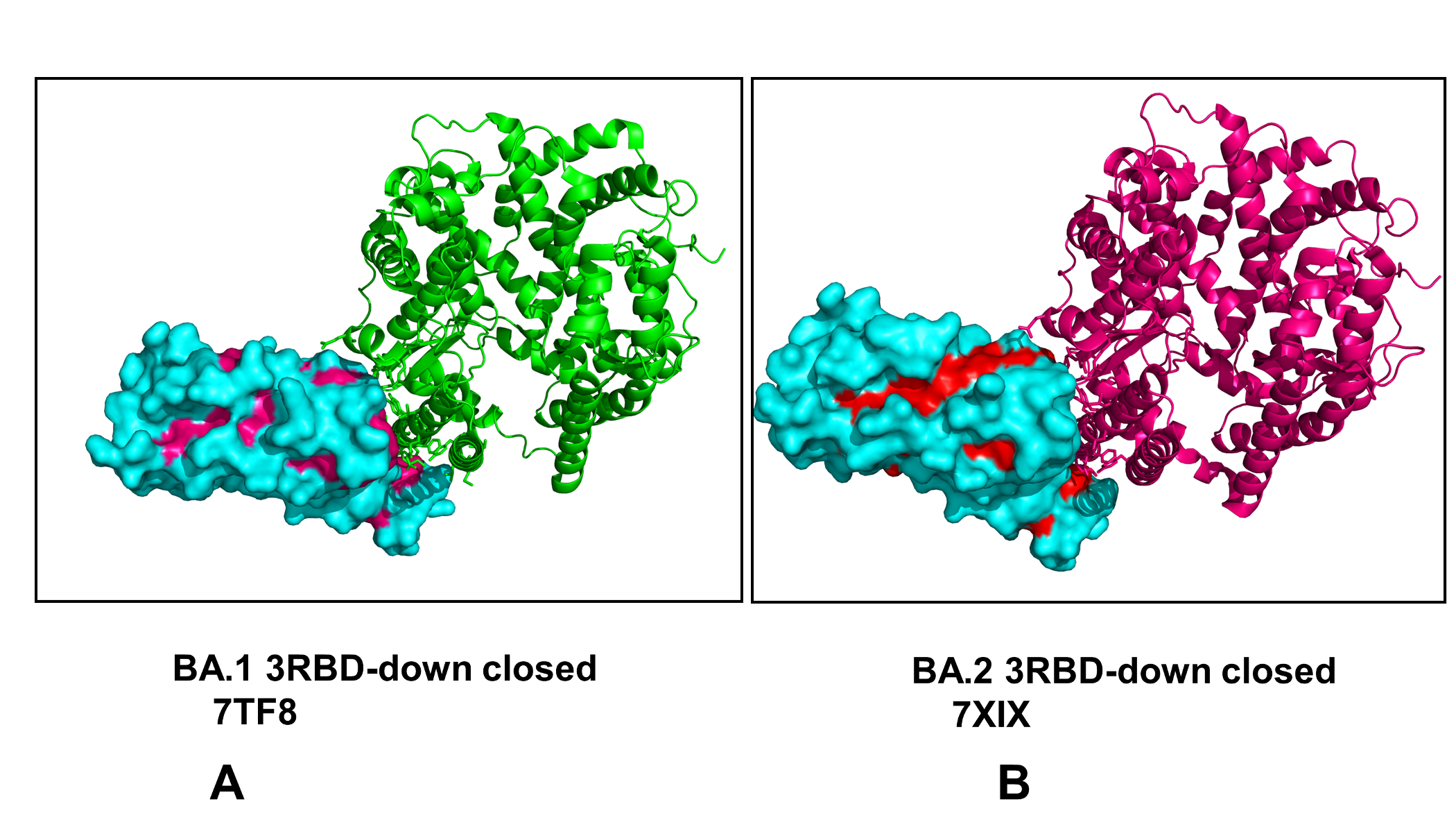

Supplement: Supplementary file 1 [file viruses-15-02009-s001.zip › SUPPLEMENTARY_MATERIALS/FigureS8.tif]

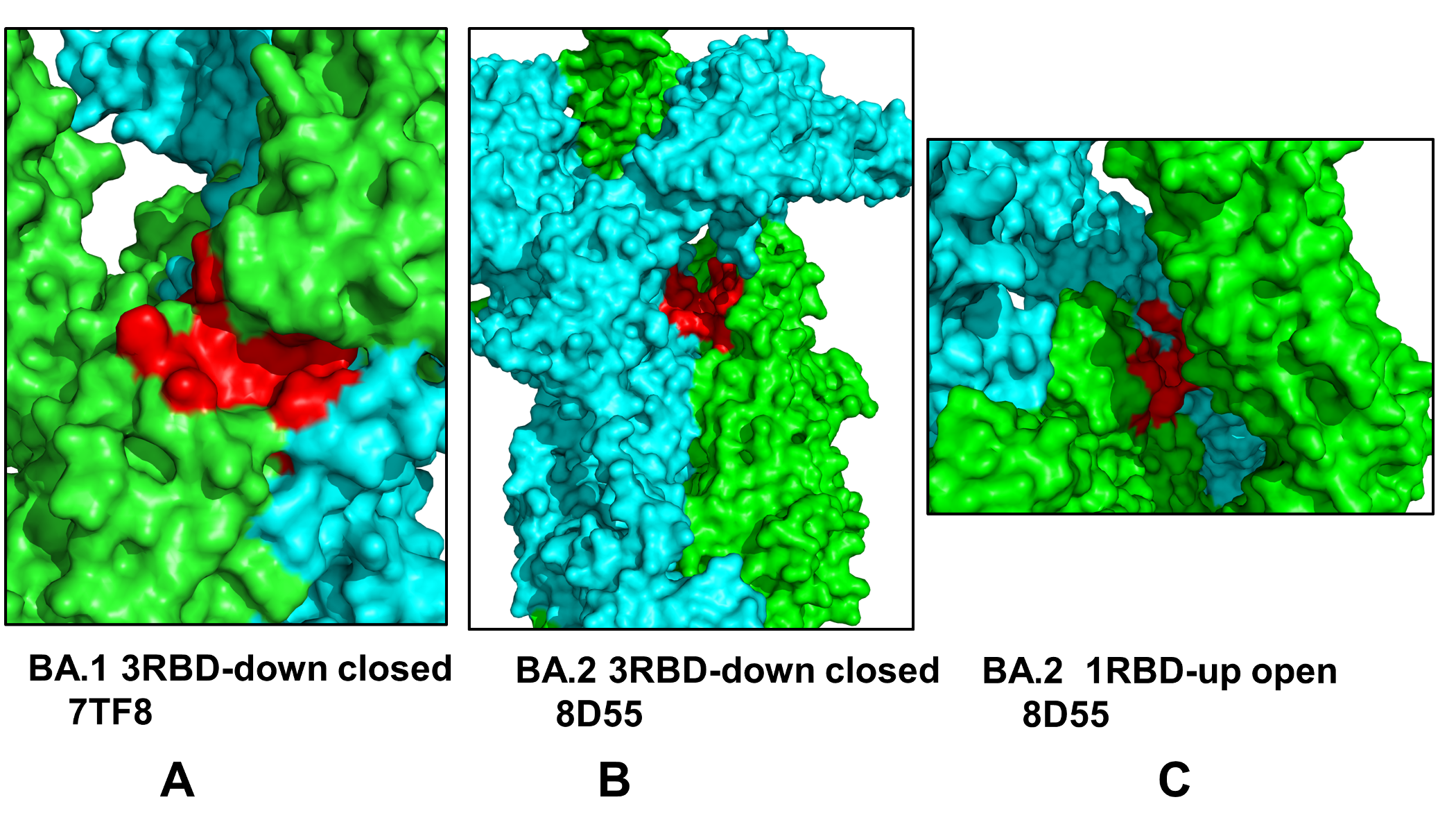

Supplement: Supplementary file 1 [file viruses-15-02009-s001.zip › SUPPLEMENTARY_MATERIALS/FigureS9.tif]
